# Abdomen—Sit

**Published:** 1883

**Authors:** 


### PART FIRST.

### COUGH SYMPTOMS.

**ABDOMEN**, **aching** in left iliac region, when walking, coughing, or raising arm; nearly takes breath away; afterward moves low down into: Eupion.

— **bleating**, cough with a bleating, as if it came out of: Cupr.

— **bruised** sensation in, on coughing: Ars.,[1] Carbo an., *Ferr.*,[1] Hyos., *Nux v.*, Plumb., Puls., *Stann.*[2]—10.

— **bruised** sensation in, on coughing and touching it: *Ferr.*[1]

— **bruised** sensation below ribs transversely, on coughing and on pressure, agg. by rising: Plumb.

— **burning** sensation in, with cough: Ars., Mez., Verat.—2.

— **bursting** sensation in, when coughing or breathing: Anac.

— **comes** from, cough seems to: Ant. cr., Sepia, Verat.

— — — epigastrium: Raph.

— **concussing**, short turns of cough concussing chest, abdomen, and occiput: Lact.[6]

— **concussion** of chest and, while coughing: Rhus,[10] Sulph.[6]

— **concussive** pain in, when coughing: Carbo an.,[10] Kali c.[6]

— **constriction** of muscles of, during coughing: Lach.

— **contraction** of, on coughing: *Chelid.*, Squil.

— **contraction** in lower, and sensation as if he would vomit, on coughing, in evening: Dros.

— **cramp** in, when coughing: Plumb.,[20] Tarent.

**ABDOMEN**, **cutting** pain in, with hollow cough in long shocks: VERAT.

— — — in lower: China.[7]

— — pain in, on coughing and deep breathing: Valer.

— **darting** in, particularly when coughing, sneezing, or touching part: Cham.[6]

— **deep** hollow cough, in three or four shocks, which seem to come from evening: VERAT.

— **diarrhœa**, sensation in abd. as from, on coughing: *Ferr.*

— **drawing in** of, with whooping cough, cough so frequent can scarcely breathe; wakes at 7 A. M.: *Dros.*[7]

— **dry** cough, as if proceeding from stomach or abd., or from constipation, or as if something remained in stomach that would not pass off: SEPIA.

— **Epigastric region**. See Stomach, and Stomach Pit.

— **griping** in, and heaving as if he would vomit, from the cough, when expectoration was incomplete or difficult: Dros.[6]

— **griping** in, when coughing: Tarent.

— **Hypochondriac region, aching** in right, at night, during cough: Sulph.

— — **bruised** feeling in, and upper abdomen, on coughing: Nux v.[10]

— — **bruised** feeling in, from cough: Bry.,[5] Carbo v., Lach.—2.

— — **contractive** pain in, with

**ABDOMEN**—(*Continued*).
cough, arresting the breathing; cough is prevented by the pain, unless he presses hand on the pit of stomach: *Dros.*[6]

— — **constriction** in, during cough: DROS.

— — **cramps** in, with cough: Zinc.[2]

— — **griping** in right, when coughing: Lyc.

— — **oppression** in, with cough: Nux v.[5]

— — **pain** in, from cough: Ambr.,[5] Amm. c., Amm. m., Arn., Ars., BRY., DROS., Hell., Hepar, Hyos.,[7] *Lach.*, *Lyc.*, Nitr. ac., NUX V.,[5] Oena. (r.), *Phos.*, Sepia, Sulph.—1 and 11.

— — **pain** in (as if forcibly constricted), when coughing: Dros.[6]

— — **pain** in, as if ulcerated, when coughing: Lach.,[6] Puls.[10]

— **Hypochondriac region, pressure** in, with cough: Acon.,[2] Ambr.,[2] Cocc., Spong., Valer.—5.

— — **stitches** in, when coughing: Acon., Amm. m., Ars., Bry., Lyc.,[2] Nitr. ac.,[2] Phos., Sabad., Samb.,[2] Sulph., Sul. ac.[2]—5.

— — **stitches** in left, when coughing: Amm. m., Bell., Carbo v., Con., Sulph., Zinc.—2.

— — **stitches** in right, when coughing: Bry., Carbo veg., Eupat. per.,[7] Kali c., Merc., Natr m., Sep.—2.

— — **stitches** in right, evening, when coughing: Sepia.

— — **support** it, must, with hands, when coughing: *Dros.*[7]

— — **tearing** pain in left, when coughing: Ambr.[2]

— — **tension** in left, during cough: Hell.[7]

— — **tension** in left, causing hacking cough: Thuja.

**ABDOMEN, Hypochondriac region, weariness** felt in, with cough: Puls.

— **Hypogastric region, bruised** feeling in, from cough: Ars., Nux v., Puls.—5.

— — **contraction** of, with cough: Dros., Squil.—5.

— — **cutting** in, from cough: Verat.[5]

— — **lancinations** in, preceding cough, as if womb would be torn off: Bell.[6]

— — **painful**, when coughing: Dros., Lyc., Nux v, Phos., Ph. ac., Sil., Squil., Verat.—5.

— — **shocks** in, when coughing: Natr. m., Squil.—5.

— — **stitches** in, with cough: Ars., Sep., Verat.—5.

— **irritation** to cough is felt in: Ambra, Ant. cr., Kali b.,[10] Sepia,[5] Thuja,[11] Verat.—2.

— **lower, bruised** sensation, on coughing: Ars.[10]

— **lower, concussive** pain in, on coughing: Carbo an.[10]

— **lower, pain** in, from coughing: Sil.

— **muscles of, bruised** sensation in, on coughing: Hyos.[10]

— **muscles of, constriction** in on coughing: Lach.

— **muscles of, contraction** in, on coughing: Squil.

— **muscles of, pain** in, on coughing: *Hyos.*, Nux v., Squil., Sulph.

— **oppression** in, when coughing: Aur.

— **pain** (undefined) in, from coughing: Alum., Ambra, Anac., Ars.,[12] *Asc. t.*, Aur.,[11] BELL.,[12] Canth., Calc., Colch.,[20] Coloc.,[20] Con., Dros., Ferr., Ipec., Kreos.,[11] Lact.,[6] *Lyc.*, Nitrum, Nux v., *Phos.*, Ph. ac., *Puls.*,[11] Rhus, Sep.,

**ABDOMEN**—(*Continued*).
Sil., Squil., Stann., Sulph.,[12] Verat.—5.
— **pain in**, and chest, with coughing, in open air: Phos.[6]
— — —, to chest on coughing: Coloc.[20]
— **pain in**, and intolerance to touch, with coughing: Cham.[2]
— **pain** all over abdomen as if raw, on coughing, violent exercise and deep breathing: Sulph.[20]
— — in sides of, and in head, from nightly cough: Lyc.[6]
— **protrude**, dry, violent cough, morning on rising, shaking, as if contents would (must support it with hands[7]): Carb. an.[6]
— **protrude**, pain in side, as if intestines would, on coughing and walking: Squil.[6]
— **racked**, and chest and occiput by short turns of dry cough: Lact.[6]
— **racking pain** in, with dry throat from violent dry cough: Squil.[6]
— **shaking** of, from cough: Kreos.[2]
— **shaking** of, as if everything would fall out, must hold abd. and sit, from severe, dry cough, in morning on rising, and nearly all day: *Carbo an.*
— **shocks** in, while coughing: Puls.[6]
— **shooting** pain in, from coughing: *Bell.*, Chin., Lach., Sep., Staph.—12.
— **sides of**, pain in, when coughing: Con., Lyc., Squil.
— — — pain in, agg. when coughing: Bor. (r).
— **side of**, pain in, as from internal wound, when coughing, blowing nose, or putting foot down; amel. by emission of flatus: Arn. (r.)

**ABDOMEN, side of, stitches** in, during day, from dry cough: Sulph.
— — — **stitches**, acute drawing, in, agg. by cough, hiccough, sneezing, or yawning: Bor. (r.)
— — — **stitches** in, agg. by cough: Carb. an. (r.), Stann. (r.)
— — — **stitches** in, on coughing: Arn. Ars.,[1] Bell. (l.), Sep.,[1] *Sulph.* (l.).—5.
— — — **stitches** in left, to small of back, when coughing: Sulph.
— **soreness** in, from coughing: Bell.,[2] Carb. an.,[2] Con.,[2] Crot. tig., Ferr., Hyos.,[12] Nux v., Puls.[10]—5.
— **soreness** in, agg. by coughing: Sulph.
— **soreness** in, on coughing or laughing: Ars.[6]
— **soreness** in lower, as from coughing: Carb. veg.
— **sticking** pain in epigastrium, must press it with hand: Phos.[7]
— **stitches** in, extending, through abdominal ring, and along spermatic cord, when coughing: *Verat.*
— See also Stomach, p. 107.
— **umbilicus**, colic as if, would be torn out, with continual cough; heat in face and sweat on forehead; after walking in open air and when lying morning and evening: *Ipec.*[6]
— **umbilicus, stitches** from, to genitals, when coughing: Sep.
— **warm**, on becoming, in, amel. cough: Sil.[5]

**ABDOMINAL RING, aching pain** in, on coughing: Nat. m.
— **pain** in, from coughing: Arn.,[5] *Cocc.*, Natr. mur., NUX V., Sil., Sulph., Verat.—11.
— **walls**, soreness in, and pain in stomach on coughing: Nux v.[7]

**ACID**, cough from nitric: Mez.[12]
— vomiting, on coughing: Natr. carb., Phos.

**ACIDS**, cough agg. by: Ant. cr., Brom., *Con.*, Lach., Natr. mur., Nux v., Sep., Sil., Sulph.—5.

**AFTERNOON**, cough in: Agar., All. c.,[12] Alum., Amm. c., Amm. m.,[11] Ant. t., Arn., Ars., Asaf., Bad.,[12] *Bell.*,[11] Bry., Caps., China, Coc. c., Gamb.,[1] Kali c., Laur., Lim.,[1] Lyc., Mag. c., Mez., Mosch., Mur. ac., Natr. c., Nux v., Phos., Stann., Staph., Sulph., Thuja,[12] Zinc.—5.
— — 1 to 2 P. M.: Ars.[5]
— — 3 P. M.: Coc. c.[5]
— — 4 to 8 P. M.: Lyc.
— **cough** in, after bath: Calc. s.
— — — agg.: Bell., Rei.
— — — from irritation in larynx: *Phos.*
— — — while sitting quietly: Coca.
— — — during siesta, from itching in upper part of larynx: Arn.
— — — touching neck: Fago.
— — — from scraping in throat, in open air: Phos.
— cough in, from tickling in throat: Bov., Mag. c
— — — in warm room: amel. in open air: Sulph.
— **deep** cough and evening, from tickling below tonsils: Amm. br.
— **dry cough** in: Kali b., Mez., Phell., Sulph., Thuja.
— — — agg. on entering warm room: Anth. n., Natr. c.
— — — from tickling at back of larynx: Anth. n.
— — — — dryness of throat: SANG.
— — — on walking: Thuja.
— **hacking cough** in: Kali c.
— — — from dryness of throat: SANG.

**AFTERNOON, inclination** to cough in: Baptisia.
— — — — agg. in: Sulph.
— — — — agg. at 7.30 P. M., on speaking: Cimic.
— **paroxysmal** cough, agg. on entering warm room: Anth. n.
— — — tickling at back of larynx: Anth. n.
— **short** cough: Chin. s.
— — — agg. in: Anac.

**AGITATED**, coughing from stitches in throat when: *Cist.*

**AGED**, cough of the: Con., Hyos.

**AIR, close**, or dust, agg. dry cough at night: Natr. ars.
— **close**, agg. hacking cough: Natr. ars.
— **cold**, excites or agg. cough: Acon., Amm. m.,[6] *Ars.*, Aur., Bar., Bry., Bov.,[10] Carb. an., Carb. veg., *Caust.*, Cepa, Cham., Cimic.,[6] Cina, Cist., Cupr., Dulc.,[6] Hepar, Hyos., Hyper.,[7] Ipec., Kali c., Kali iod.,[10] *Lach.*, Mez., Nitr. ac.,[6] Nux m., Nux v., *Phos.*, Phos. ac., Rhus, Rumex, Sabad.,[6] Sac. alb.,[1] Samb., Sep., Sil., Spong., Squil.,[6] Stram., Sulph.—2.
— **cold**, amel. cough: Coc. c.
— **icy cold**, seems to stream through air-passages on deep inspiration, with desire to cough: *Coral.*
— **cold**, persistent coughing after walking in, also when lying down, excited by deep inspiration, accompanied by colic, as if umbilicus would be torn out, heat in face, and sweat on forehead: Ipec.[6]
— **damp cold**, agg. cough: Ant. t., Calc., Carb. an., Carbo veg., Chin., Dulc., Lach., Mag. c., Mosch., Mur. ac., Nitr. ac., Sulph., Sul. ac., Verat., Zinc.—2.

**AIR**, **draught** of, agg. cough: Acon., Caps.,[7] Caust., China.—2.
— **dry cold**, agg. cough: Acon., Cham.,[2] Samb.,[2] Brom., Phosph., Hepar, Nux m., Spong.—10.
— **entering cold**, agg. cough: *Carb. veg.*, Phos.
— **inspiring cold**, agg. cough: *Cepa*, Cist., *Cupr.*, Rumex,[2] Staph.,[2] Vit.—5.
— **inspiring cold**, causes hacking cough: CEPA,[1] Phosph.[10]
— **walking in cold**, agg. cough: Ars., Ipec., Phosph.,[10] Verat.[7]—1.
— **warm room**, going from, to cold air, or *vice versa*, causes coughing: Sepia, Nux v.,[10] Natr. c.[10]
— **night**, agg. cough: Merc.
— **open**, excites or agg. cough: Acon.,[12] Alum.,[11] Ang., ARS., Bar., Bry., Calc., Carbo veg., Cham., Cina, Cocc., Coff.,[7] Dig., Ferr.,[12] Ipec., Kali b., *Lach.*,[11] Lyc., Mosch., Nitr. ac., NITRUM,[2] Nux v., Osm., *Phos.*, Phos. ac.,[1] Rhus, Rumex, Seneg., Sil., Spig., Staph., Stram.,[12] *Sulph.*,[12] *Sul. ac.*[7]—5.
— **open**, coughing in, with pain in abdomen and chest: Phos.[6]
— — at night, causes cough: Calc. ph., Linu., Phos., Spig., Sulph., Sul. ac, Trif. p.
— — amel cough: Dulc., Nux v.,[5] Sulph.[1]
— — amel. dry cough: Iod.[1]
— — causes dry cough: Spig.
— — entering, agg. cough: *Acon.*, Bry., Ipec., Rumex, Squil.—12.
— — agg. hacking cough: Seneg., Sulph.
— — amel. hacking cough: Lil. tig.
— — amel. rough cough: Iod.
— — short cough from: Spig.
— — agg. tickling cough: *Lach.*
— — going into warm room from,

**AIR**—(*Continued*).
excites cough: Acon.,[12] Ant. cr.,[7] Bov.,[1] Brom.,[7] Coc. c.,[10] Natr. c.,[10] Sep.[1] Sulph.,[1] Verat.[10]
— — on entering warm room from cold, feels sensation in trachea as if full of smoke, which excites cough; feels as if he could not inhale sufficient air: *Bry.*[6]

**AIR PASSAGES**, burning in, with cough: Ant. cr., Carb. veg., Caust., Cina, Iod., Lach., Mag. m., *Spong.*, Sulph., Zinc.—11.
— — **catarrh of**, dry cough at night in bed, as if from: Coca.
— — **crawling** in, at night, causes hacking cough: Æth.
— — **crawling** irritation near suprasternal fossa, before midnight, causes cough, agg. by swallowing mucus: Apis.
— — **dryness** of, causing cough: Carb. an., Lach., Merc., Petrol., Puls.—11.
— — **dull cutting**, from below upward in, which becomes a stitch and excites two or three fits of coughing: *Arg.*
— — **irritation** in, causing cough: Ars., Colch., Kali b.
— — **irritation** in, causing hacking cough at night: Kali b.
— — **irritation** in, to cough: Chlo.
— — **irritation** in, when coughing: All. s.
— — **irritation** in, evening, causes cough: Sulph.
— — **irritation** in, evening, in bed, causes cough: Agnus, Amm. c., Coff., Kali c.
— — **irritation** in, low down, causing coughing at night: Cham.
— — **tickling irritation** in, causing cough: Ars., Calc. ac.,[1] HYOS.,[1] Kali b., Nux v., Puls., Staph., Verat.—10.
— — **tickling irritation** in, as if

**AIR PASSAGES**—(*Continued*).
it would provoke cough, makes breath short, and is amel. by moderate exertion: *Rhus.*

— — **tickling irritation** in small spot: Apis, *Con.*—10.

— — **irritation** in, upper part, causing coughing: Plan.

— — **mucus** gets into, on stooping, or going up-stairs, which is expelled by a single cough: *Arg.*

— — **pain** in, agg. when coughing: Camph.

— — **raw**, pain as if, with cough: Ambr., Anac., Ant. cr., *Arg.*, Arn., Calc., Carb. veg., Grat., Kali c., Kreos., Laur., Mag. c., Mez., Nux mos., Petrol., *Phos.*, Ruta, Sep., Sil.—11.

— — **rawness** of, causes cough: *Coc. c.*, Stann.

— — **rawness** of, causes coughing, 10 A. M., while lying: Coc. c.

— — **sore**, pain as if, with cough: Alum., Ambr., Amm. c., Arg., Ars., Baryt., *Bell.*, Calc., CARB. VEG., *Caust.*, Chin., Cina, Hep., Lach., Lycop., Mag. m., Merc., Natr. c., Nux mos., Nux vom., PHOS., Sep., Sil., Spig., Spong., STANN., SULPH.—11.

— — **stitches** in, with cough: Kali c., Lach., Phosp.—10.

— — **stitches** in, extending upward, causing cough: *Arg.*

— — **tickling** in, causing cough: ACON., Amm. c., Amm. m., Angus., Ant. t., Arg. n., *Arn.*, Asaf., Bar., Bell., Bor., Bov., *Brom.*, Bry., Caps., *Carb. an.*, *Carb. veg.*, *Caust.*, CHAM., Chin., Cina, Coc. c.,[1] Colch., *Con.*, Cupr., Dig., Ferr., Graph., Hepar, Ign., IOD., IPEC., *Kali b.*, Kali c., LACH., Lact., Laur., Led., Mag. c., Mag. m., Marum, Merc., Mur. ac., Natr. c., NATR. M., Nitrum, NUX VOM., Olean., *Phos.*, Prun., *Puls.*, Rumex,[10] Sabin., Sant.,[1] Seneg., SEP., Sil., *Spong.*, *Stann.*, STAPH., Sulph., Verat., Zinc.—11.

— — **tickling** in, at night, causing cough: Rumex,[10] Sant.[1]

— — **tickling irritation** in, causing cough: Calc. ac., *Hyos.*

— — **tickling** low down in, causing cough: Cina.

— — **wall, posterior**, of, **tickling** low down in, causing cough, agg. by lying and sleeping, amel. by loosening mucus: Apis.

**AIR**, warm, agg. coughing: Ant. cr., Cocc. c.,[10] Iod.—12.

— with headache, cough: Lach.

**ANGER**, coughing **from**: Acon., *Ant. t.*, Arg. n., Ars., *Cham.*, Chin., *Ign.*, Nux v., Sep., *Staph.*, Verat.—2.

— **before** coughing: Asarum.

— **from** the cough: Acon.,[5] Ant. t., Arn., Bell., Cham.—6.

— with **fright**: Acon., Ign.—2.

**ANGRY, when getting**, violent spell of coughing comes on: *Ant. t.*, Arg. nit.,[7] Arn., Asar., Cham. —8.

— coughing if child gets, vomits food and mucus: *Ant. t.*[2]

**ANGUISH**, accompanying coughing: *Acon.*, Cina, *Coff.*, *Dig.*, Hepar, Iod., Rhus.—11.

**ANUS**, pain in, with coughing: *Lach.*[6]

— **spasmodic** constriction of, with violent dry cough: Acon.[6]

**ANXIOUS**, cough: Ars., Cina. Coff., Rhus.—5.

**ANXIOUS**, **before** attack of whooping cough: Cupr.[8]
— cough before midnight, on waking: *Rhus.*

**ANXIOUSNESS**, obstruction of breath, pale face and whimpering, cough ending with: Cina.

**ANXIETY**, with cough: *Acon.*, Cina, *Coff.*, Dros., Eupat. per., *Hepar*, Iod.,[12] Rhus, Samb., Spong., Stram.—5.
— **nocturnal**, with cough: *Acon.*[12]

**APHONIA**, cough with: Amm. caust.,[10] Phos.,[12] Spong.[10]

**APPETITE**, cough, with loss of: Podoph.

**APPREHENSION**, . **discouragement** and, following short cough, caused by severe tickling and irritation behind upper sternum: RHUS.[1]

**ARMS**, **becoming cold**, cough from: Ars., Calc., Ferr., *Hepar*,[5] Kali c., *Rhus*,[7] Sil.[2]—12.
— **moving**, agg. cough: Ars., Calc., Ferr., Kali c., Led., Lyc. (r.),[12] Natr. mur.[5]
— **putting out of bed**, or becoming cold, causes coughing: HEPAR, RHUS, Sil.
— **stretching out**, causes coughing: Lyc.[5]
— See also under Limbs.

**ARTERIES**, and also **heart**, violent beating of, from dry cough, before midnight: Calc.[6]

**ARTHRITIC** pains brought on by least cold, with irritable cough; swallowing excites pains, fear of least touch: Ph. ac.

**ARTICULAR** pains increase, cough amel. as: Coloc.

**ASCARIDES**, spasmodic coughing in persons troubled by: Mag. s.[14]

**ASCENDING**, cough agg. on: Arg. n.,[7] Ars., Bar., Iod., Lyc., Mag. c., Mag. m., Merc., Nitrum, Nux vom., Seneg., Sep., Spong., Squil., Stann., Staph., Zinc.—5.

**ASLEEP**, **before getting**, coughing: Calc.,[10] Merc.[2]
— **on falling**, at night, coughing: Lach.,[10] Petrol. (v. sleep.)

**ASTHMA**. See Supplement.

**ASTHMATIC** affections (dyspnœa, obstructed respiration, etc.), with cough: *Acon.*, *Alum.*, Amm. c., Amm. m., Anac., ANT. T., Aralia,[10] Arg. n., Arn., ARS., Aspar., Bar., *Bell.*, *Brom.*, Bry., Calad, Calc., Carbo an., Carbo v.,[2] Caust., China,[2] Cic.,[6] CINA, Coc. c.,[10] Cocc., Con., Coral.,[10] CUPR., Dig.,[2] Dolich., DROS.,[2] Euphr., Ferr., Guai., *Hepar*,[11] Ign.,[2] Iod.,[6] IPEC., Kali b.,[6] Kali c.,[2] Kali chl.,[6] *Kreos.*, *Lach.*, Lact.,[10] Laur.,[2] *Led.*,[12] Lyc., Merc., Mez., Mur. ac., Natr. m.,[2] Natr. s., Nicc., Nitr. ac., Nitrum, *Nux m.*, NUX v., OP., Phell., Phos., Puls., Rhus, Samb.,[11] Sang.,[6] *Sep.*,[2] *Sil.*,[2] Spig., Spong., Squil., Stann., Sulph., Sul. ac.,[2] Verat., Viola od.,[7] Zinc.,[2] Zing.[10]—12 and 16.

See also Choking, Suffocative Coughs.

— cough: Acon., Amm. c., Ars., Asaf.,[2] Bar., Bell., Carbo v., Cham.,[2] Euph., Hepar, Iod., Ipec., Laur., Kali c., Lob., Lyc., Mosch., Nux v., Petro., Phos., Sabad.,[2] Samb., Sepia, Spong.,[7] Stram., Sulph., Sul. ac.[2]—5.

**ATMOSPHERE**, **from change** of, coughing: Erio., Rumex.
— **stormy**, agg. coughing: Phos., Rhod.,[10] Sil.—5.

**ATTACKS. See Paroxysms.**

**AUTUMNAL** cough: Verat.[12]

**AWAKING.** See Waking, Wakening.

**BACK, aching in,** on coughing: Amm. c.,[10] Merc., Narz.,[1] Nitr.,[1] Puls.[10]

— **click down,** during coughing: Apis.

— **cramp-like stitch,** to hypochondria, lying or coughing, agg. by standing or sitting: China.

— **and limbs** stiff, with hard, tickling cough; worse evenings till midnight, on lying: Rhus.[7]

— **lower,** pressure in, to testicles, on coughing: Osm.

— **lower** stitches in, on coughing: Caps.,[10] Nitr. ac.,[1] Sep.,[10] Sulph.[6]

— **lumbar region,** aching in, during coughing: Arund., Merc.

— **lumbar region,** aching in, agg. by cough: Nitr.

— **lumbar region,** pain as if hernia would protrude, dares not cough or inhale: Sul ac.[6]

— **lumbar region,** stitches in, chest, hip, and uterus; pain in the sternum, with tightness of chest and rattling of mucus in chest, with cough: Bell.[10]

— **lumbo-sacral** region, pain in, agg. by coughing and bending: Phos.

— **lungs,** sensation as if, touched the, when coughing: Sulph.[6]

— **lying on,** agg. cough: Amm. m., Iod.,[10] Nux v., Phos., Plumb.,[7] Rhod.,[10] Rhus,[10] Sil.—5.

— **lying on,** agg. dry cough: *Phos.*

— **lying on,** irritation to cough: Natr. m.[1]

— **lying on,** midnight, dry cough from: *Nux v.*

**BACK, lying on,** dry cough, going off when turning on side: Nux v.[6]

— **lying on,** relieves cough: *Mang.*

— — — partially relieves dry cough, with nervous excitability: *Acon.*[7]

— **lying on, pain** in, or right side, agg. irritable cough: Phos.

— **midsternum** through to, cough with pain from: *Kali b.*, Rumex.[10]

— **pain** in, from coughing: Cocc., Bry., Kreos.,[5] Nitrum.[10]—12.

— **pain** in, and chest, cough sounds as if whole inside of chest were dry, with a: *Merc.*[6]

— **pain** in chest, compelling him to lie on, with violent cough: *Acon.*

— **pain** from midsternum through to, with cough (and raising of tough black mucus[6]): *Kali b.*[1]

— **painful jerks** in, and in ossa ischii, on sneezing or coughing: *Nux v.*[6]

— **pain** between, or in region of scapulæ, worse from sneezing, gaping, coughing, or any jar: Calc.[7]

— **pain** in small of back, with cough: *Amm. c.*, *Kali b.*, Merc., Nitr. ac., Sulph.—11.

— **pressure,** violent, from midsternum to, with sensation as of a stone pressing downward in pleural cavities, causing painful cough: Coral.[6]

— **shootings** in, with cough: Merc., Puls.,[11] Sep.[16]

— **shoulders,** pain between, during coughing: Stram.

— **small of, pain** in, with cough: *Amm. c.*, *Kali b.*, Merc., Nitr. ac., Sulph.—11.

Page(s) missing

**FACE, redness**, circumscribed, of cheeks: Samb.[2]
— — — — — with yellow complexion: Lyc.[2]
— — — — — with febrile excitement in afternoon, with dry or fatiguing cough: Sang.[2]
— **rubbing** the, children: Squil.[5]
— **shocks** upon the zygoma: Hepar.[2]
— **sunken** look: Chin., Cupr., Stann.—2.
— **sweat**. See Perspiration.
— **tension** of the: Verb.[2]
— **twitching** in the: Ant. t., Spong.—5.
— — in facial muscles: Cham.[2]
— **wild** look: Bell.[5]
— **wrinkles** in: Lyc., Stam.—5.
— **yellow**: Caust.,[5] Lyc., Mag. m., Puls., Sep., Verat.—2.
— — around eyes: Nitr. ac., Nux v.—2.
— — around mouth: Nux v., Sep.–2.
— — around nose: Nux v.[2]
— — across nose and cheeks: Sep.[2]

**FAINT HEARTEDNESS** with cough: Bar., Sil.—2.

**FAINTING** and wheezing precede the cough: Kali c.[2]

**FAINTNESS** during cough: Coff.,[1] Lach., Op.—5.

**FALLING** down during cough: Ipec.[2]

**FASTING** agg. cough: Kali c., Mag. m.—2.

**FAT** food or fruit, agg. cough: Mag. m.[2]

**FATIGUING** cough. See Exhausting.
— — day and night, agg. by motion, with scanty expectoration: Sil.[6]

**FAUCES, caustic** sensation in, exciting convulsive cough: Coll.
— **choking** in upper part of, with cough: Cocc.[5]

**FAUCES, constriction** in, excites cough: Æsc. hip.
— — — — dry, hacking cough: Æsc. hip.
— **dryness** of, excites dry cough: *Mez., Phyt.*
— — — — hacking cough: *Phyt.*
— — roughness and scraping deep in, excite hacking cough: DROS.
— **irritation** in, excites cough: Dios., Lycopers., Mag. s., *Mez.*, Sul. ac.
— — — excites dry cough: *Mez.*
— — — — hacking cough: Dios., Mag. s.
— **isthmus, of scraping** in, causing cough on inspiration: Nitrum.
— — — **trickling** in, provoking cough: Tilia.
— **pain** in, on coughing: Caps., Chin. s.
— **rough**, scraping, dry sensation in, causing hacking cough, yellow mucus expectoration, and hoarseness, so that he can only speak with great exertion, in a deep bass voice, together with great oppression of the chest, as if the air were withheld on talking and coughing, so that the breath could not be expired: DROS.
— **roughness**, dry, causing cough: Gels.
— **scraping** in, on inspiration, causing cough: Kali b.
— **soreness**, on coughing and blowing nose: Carb. veg.
— **tearing** in, during cough: Chin. s.
— **tickling** in, causing cough: Carb. ac., Gels., Lact.
— — — causing inclination to cough: Tilia.
— — — from irritation to cough: Aloe.
— ulcerative, tickling sensation of, causing cough: Sars.

**FEARS**, to cough, and seems to avoid it as long as possible—children with bronchial catarrh: Phos.

**FEATHER**, or corn of barley, sensation of, in one of the bronchia, with sensation as if it were swaying to and fro with the respiration, excites cough: Rumex.[2]

— dust, sensation as from, in throat, excites cough: Amm. c., Bell., Calc., *Chin.*, Cina, Cycl., *Ign.*, Marum., Meph.,[10] Puls.—11.

**FEATURES distorted** (twitching) with cough: Ant. t., Spong.

**FEBRILE attack**, exhausting, cough during: Thuja.

— — during, short cough, cold hands and feet, wants fire in room: Carb. v. (Hg.)

— — during, short breathing after each cough: Phos. (Hg.)

— — during breathing hard with cough: Nitr. ac. (Hg.)

**FEET, aching** in, sleep is disturbed by frequent coughing and: Sepia.[6]

— cold, agg. cough: Bar.[5]

**FEVER** (heat) **agg.** by coughing: Ambr., Amm. c., Ant. t., *Arn.*, *Ars.*, Bell., Carb. v., Hepar, Hyos., Iod., Ipec., Kreos.,[11] Lach.,[11] Led., Lyc., Mag. m., Natr. c., Nux v., Phos., Puls., Sabad., Squil., Sulph.—3.

— **cough** with expectoration during: Alum., Arg., ARS., Bell., Bism., Bry., CALC., Carb. v., Cic., *Chin.*, Dig., Dros., Dulc., Ferr., Iod., KALI C., Ruta, Seneg., Sep., *Sil.*, Spong., Squil., Stann., Staph., *Sulph.*, Thuja.—3.

— **cough** without expectoration, during: ACON., Ang., Ant. cr., *Apis*, *Arn.*, *Ars.*, Bell., Brom., *Bry.*, Calc., Carb. v., Caust., Cham., Cina, Chin., Coff., CON., Cupr., Dros., Hepar, *Hyos.*, Ign., IPEC., *Kali c.*, Lach., Lyc., NATR. M., Nitr. ac., Nux m., NUX V., Op., Petro., PHOS., Plat., Puls., Rhus, SABAD., Samb., Sep., Spig., Spong., Squil., Staph., Sulph., Sul. ac., Tarent.,[1] Verat., Verb.—3.

— **during**, agg. of cough: Lach., Nitr. ac., Seneg.—5.

— **remittent**, cough during: Podoph.[5]

— **suppressed** intermittent, hectic cough from: Eup. per.[10]

— **scarlet**, cough after: Ant. cr., Con., Hyos.—5.

— **typhoid**, cough during: Ant. t., Bell., Bry., Calc., Lyc., Nitr. ac., Phos., Rhus, Sang.—5.

**FINGER**, cough as from, thrust into back of mouth: Merc. cor.

**FIRE**, looking into causes cough: *Ant. cr.*

**FITS** of cough. See Paroxysms.

**FLATUS**, dry cough wakes patient, must sit up; ceases only when sits up and passes flatus up and downward: SANG.[6]

**FLUIDS**, cough seems to be from an increased secretion of, in the larynx: Lach.

— cough from loss of animal: Chin., Cina, Ferr., Ph. ac., Staph.—5.

**FLUTTERING** cough from tickling in larynx: Lach.

**FOOD**. See under Eating and Drinking.

**FORCIBLE cough**: Acon., Alum.,[2] Bry., Con., Hepar, *Phos.*

— — **amel.** by expectoration of tenacious mucus: *Phos.*

— — **evening** and night: Ruta.

— — from tickling and itching in larynx: Lyc.

**FOREIGN** body in larynx seems to excite cough: Bell.[11]

**FOREHEAD, pain** in, from cough: Asc. t., Kali b.[6]
— **pressure** on left side of, when coughing: Coca.
— **pulsation** in, during cough: Hipp.
— **sensation** as if something would fall out of, during cough: Hepar.
— **stitches** in, on coughing: Anac., Arn., Sulph.
— — — — — at midnight: Hepar.
— — — agg. by cough: Ant. t.
— — above eyes, when coughing: Hyos.
— See also under Head.

**FORENOON**, cough in the: Agar.,[1] Alum., Amm. c., Amm. m., *Bell.*, Bry., Canc.,[1] Chin. s.,[1] Coc. c., Hell.,[1] Kali c., Mag. c., Natr. c.,[1] Natr. m., Rhus,[11] Sabad., Sars.,[1] Seneg., Sep., Sil., Stann., Staph., Sul. ac.—5.
— **cough** at 1 A. M.: Coc. c.
— — — 1–2 A. M., from scratching in larynx: Zing.
— — — 1–4 A. M., prickling in larynx: Bufo.
— — — 2 A. M.: Phos., Cocc., Rum.
— — — 2 or 3 A. M.: Ant. t., Merc.
— — — 3 A. M.: *Amm.* c., Bapt., Cupr., KALI C., Mag. c., Mur. ac., Nitrum.
— — — 3–4 A. M.: *Amm. c.*, Lyc.
— — — 4 A. M.: Anac., Nitr. ac., Nux v.
— — — 5 A. M.: Arum. t., Kali c., Rum.
— — — 6–7 A. M.: Arum. t., Calc. ph.
— — — 8 A. M.: Ham., Ol. an.
— — — 8–9 A. M.: Sil.
— — — 9 A. M.: Sep., Tarent.
— — — 10 A. M., from rawness in air passages while lying: Coc. c.
— — — 10–12 A. M.: Natr. m.[5]
— — — 11 A. M., from rush of blood to chest: Raph.

**FORENOON, cough, agg.**: Natr. ars.
— — — 9–12 A. M.: Staph.
— — — 10–12 A. M.: Coc. c.
— — **air passages**, rawness of, cause cough at 10 A. M., while lying: Coc. c.
— — **amel.** on sitting up: Euphr.
— — **chest**, from rush of blood to, at 11 A. M.: Raph.
— — **larynx**, from prickling in, 1–4 A. M.: Bufo.
— — — — scratching in, 1–2 A. M.: Zing.
— — **throat**, from tickling as by a feather in: Glon.
— — — — — low down in: Dios.
— — — — sweetish taste in, while lying: Mag. c.
— — **waking**, on: Dios., Natr. m.
— — — after: Rhus.
— **convulsive** cough: Agar., Lact.
— **dry** cough: Agar., Alum., Grat.
— — — 1–2 A. M., from scratching in larynx: Zing.
— — — 2 A. M.: *Opium.*
— — — 3 A. M., tickling and scraping in larynx: Op.
— — — 11 A. M., tickling behind upper half of sternum, when sitting bent forward: *Rhus.*
— — — **agg.** in: Camph.
— — — **larynx**, from scratching in, 1–2 A. M.: Zing.
— — — — — tickling and scraping in, 3 A. M.: Op.
— — — **sternum**, tickling behind upper half of, when sitting bent forward, 11 A. M.: *Rhus.*
— — — **throat**, from roughness in: Sars.
— — — — — tickling in: *Amm. m.*
— — — **trachea**, from irritation in: Coc. c.
— **hacking** cough, from tickling in throat: *Amm. m.*

**FORENOON, paroxysmal** cough: Coc. c., Grat.

— — — 2–3.30 A. M.: Coc. c.

— — — 10 A. M.: Aqu. fr.

— **short** cough: Agar., Alum.

— — — 7 A. M., on waking: Dign.

— — — 11 A. M., aching behind upper sternum, on sitting bent forward: Rhus.

— — — after **siesta**, from irritation and tickling behind sternum: Rhus.

— — — **sternum**, aching behind upper, on sitting bent forward, 11 A. M.: Rhus.

— — — **trachea**, from irritation in: Coc. c.

— — — **on waking**, 7 A. M.: Dign.

**FRIGHT** *after*, coughs when lying down, stitches in pit of stomach: Rhus.[8]

— after, dry, spasmodic cough: Bell.[8]

— — suffocation, vomiting, or coughing, bluish face, whining, and trembling: Samb.[8]

— — loses breath after a little cough; after vomiting, coughing: Samb.[8]

— — whooping cough: Acon., Ign., Stram.—8.

— with *vexation*, agg. cough: Acon.[5]

**FRUIT** or fat food, agg. cough: Mag. m.[2]

See under Eating.

**GAGGING** cough, morning after rising: Cina,[7] Sepia.

— belching or vomiting with cough: Cimex.[7]

— hoarse cough, evening: Cina.[7]

**GAPING** after attack of cough: Anac.[6]

— and coughing consecutively: Ant. t.[7]

— excites cough: Arn.,[6] Asaf.[7]

**GARMENTS**, removing outer, cough from: Cer. b.

See Undressing.

**GASPING** for breath before cough: Ant. t.,[7] Brom.,[5] Bry.,[6] Cocc., Coral.[7]

— — — See under Breathing.

**GASTRIC** cough: Kali ars.

**GAYETY** with cough: Verat.

**GLANDS**, sub maxillary, swelling of with coryza and cough, pain in throat during deglutition; great chilliness: Silicea.[6]

**GLISTENING** object, agg. of cough from: Stram.[7]

**GONORRHŒA**, long continued dry cough after suppressed: Benz. ac.[10]

**GRASPING** at throat by child every time it coughs: Acon.[7]

— larynx involuntarily at every cough: feels as though larynx would be torn: All. c.[7]

**GRIEF** and care agg. the cough: Phos. ac.

— or sorrow, whooping cough from: Phos.[8] Cham.[8]

**GRIEF** or sorrow, cough from in children: Arn., Asar., Cham.[8]

**HACKING cough**: Acon.,[5] Aeth., Agar., Alco., *Aloe.*, ALUM., Am. c.,[5] Am. m., Aml. n., Anac.,[5] Ang., Ant. cr., Arg. n., Arn., Ars., *Ars. iod.*, Asaf., Asar.,[5] Asc. t., Benz., Bov., Brom., *Bry.*, Calc., Camph., Cann. i., Cann. s., Canth., Caps., Carb. ac., Carbo v., Caust., Cham., Chin.,[5] Chro. ac., Cimic., Clem., Cob., Coc. c., Coff., Colch.,[5] Coloc.,[5] Con., *Cupr. s.*, Cycl., Dign., Dros.,[5] Dulc., Eupat. per.,[2] Euph.,[5] Eupion, Fran., Gels., Grat., Guare., Hell., Hell. ox., Hcy. ac.,[5] *Hyos.*, *Hyper.*, Ipec., Jatr., Kali b., Kali c.,[5] Kali mag., Lach., Lauro., Linu., Lyc., Mag. s.,

**HACKING cough** (*Continued*). Merc. sol., Merc. p. iod.,[5] Mur. ac., Natr. ars., Natr. c, Natr. m., Nicc., Nitr. ac., Nitrum, Ol. an., Ol. j., Op.,[5] Osm., *Par.*, PHOS., Phyt., Plumb., Pte., Ran. sc., *Rhus*, *Rumex*, SANG., Seneg., *Sep.*,[10] Sil., Sin. n., Squil., Stann., Sticta,[2] *Stil.*, Stront, Sulph., Sul. ac, Sum., Thuja, Til., Trif. p., Ust., Val., Xan., Zinc,[5] Zing.—1.

— — **afternoon**: Kali c.

— — — from dryness of throat: SANG.

— — **air**, close, agg. in: Natr. ars.

— — — open, agg. in: Seneg., Sulph.

— — — — amel. in: Lil. t.

— — **air-passages**, from crawling in: Aeth.

— — — — irritation in: Kali b.

— — — — smothered feeling in: Asaf.

— — — — tickling from pharynx to: Hcy. ac.

— — **bed**, evening in: Bry., Lact., Nitr. ac., *Rhus*, SEP., Sulph.

— — **breathing**, arrest of, from dry, hacking cough, with vomiting: ALUM.

— — **bronchi**, from irritation in: Trif. p.

— — **chest**, from pressure in: Op.

— — — rough, scraping dry sensation in fauces, causing hacking cough, yellow mucus expectoration and hoarseness, so that he can only speak, with great exertion, in a deep bass voice, together with oppression of chest as if air were withheld on talking or coughing, so that breath could not be expired: DROS.

— — — from rawness in: Osm.

— — — soreness of, from dry, hacking cough: Sil.[6]

— — — from tension in left: Thuja.

**HACKING cough, chest**, tickling under middle of sternum causing hacking cough, worse from moving or talking: Calc. (C. Hg.)

— — during **day**: Gamb., Natr. m., *Sum.*

— — — **chill**: Calc. ph.

— — after **dinner**: Agar., HEPAR.

— — **dust**, agg. from: Natr. ars.

— — **eating**, while: Anac., *Hepar* Nitr. ac.

— — — — agg.: Sang.

— — **epiglottis**, from tickling in Acon., Tarax.,[10] Wye.

— — **evening**: Alum., Amm. br., *Bor.*, Carb. an., Coloc., Dign., Eupat per., Kali b., Ol. an., Phos., Sep., Sil. t., Sin. n., Stront., Sum. Zinc.

— — — 2 P. M.: Laur.

— — — 3 P. M.: Calc. ph.

— — — 3–4 P. M., agg.: Calc. f.

— — — 6 P. M.: Sum.

— — — 7 P. M., from tickling in trachea, on entering warm room: Com.

— — — **agg.** in: Phos.

— — — in **bed**: Bry., Lact., Nitr. ac., *Rhus*, SEP., Sulph.

— — **fauces**, from constriction in fauces: Aesc. h.

— — — — dryness in: *Phyt.*

— — — — dryness, roughness, and scraping deep in: DROS.

— — — — irritation in: Dios., Mag. s.

— — — — roughness, scraping, and dryness deep in: DROS.

— — — — tickling in: Tilia.

— — **forenoon**, from tickling in throat: Amm. m.

— — **hypochondrium**, from tension in left: Thuja.

— — **inspiration**, on: Merc. i. fl.

— — — of cold air: ALL. C.

— — — deep, agg. on: Natr. ars.

**HACKING cough, inspiration,** deep, amel. on: Osm.
— — **irritation** to: Ang., Cham., Mag. s., Natr. ars., *Stann.*
— — **larynx**, from crawling in: *Dros.*, Psor.
— — — — irritation in: Osm., Sac. alb., *Seneg.*
— — — to, felt in, evening, after lying down: IGN., Kali b.[10]
— — — from itching in: Laur.
— — — — itching—tickling in: Calc. f.
— — — — mucus in: Cocc., Seneg.
— — — — provocation in, evening, after lying down: IGN.
— — — — tenacious mucus in: Dig.
— — — — tenacious mucus, which seems to be firmly adherent to the posterior part of the: *Par.*
— — — — scratching in: Zing.
— — — — tickling in: Acon.,[10] *All. c.*, Ang.,[10] Bell.,[10] Carb. an., Coc. c., Dig.,[10] Ipec., Lach., Laur., Lob. s., Lyc., Rumex, Sabad.,[10] Spira., Spong.[10]
— — — — tickling, felt only when walking in open air: Ang.
— — — — sensation of thickening of mucous membrane of, with yellow, stringy expectoration: Ery. aqu.
— — — — small spot, tickling in: Apis, Cimic., Con.—10.
— — **lying down**, when: Par., Vesp.
— — — — agg: Sulph.
— — — — evening, after: SANG.
— — — — evening, after, agg.: Sil.
— — — evening, amel.: Amm. m.
— — — — or left side: Par.
— — — — from provocation in larynx: IGN.
— — **menses**, at beginning of: Phos.

**HACKING cough, morning:** All. c., Calc., Cina, Ir. v., Kali c., Kali iod., Mit., Nitr. ac., Ol. an., Sil., Sum.
— — — agg. in: Sum.
— — — **rising**, after: Arg., Par.
— — — from mucus: Laur.
— — **night**: Graph., Kali c., Mag. s., Natr. m.
— — from **mucus**, morning: Laur.
— — — — in mouth: Sul. ac.
— — **palate**, from dryness, roughness, and scraping in, soft: DROS.
— — — — tickling in: Tilia.
— — — — tickling in pharynx and, noon: Arg. n.
— — **pharynx**, from irritation in: Trif. p.
— — — — tickling in: Mag. s.
— — — to air-passages, tickling from: Hcy. ac.
— — — tickling in, night: *Sil.*
— — — tickling in palate, etc., noon: Arg. n.
— — **respiration**, from tightness of: Nux v.
— — **ribs**, from teasing sensation beneath the right fourth and fifth ribs: Natr. ars.
— — **rising**, after: Benz. ac.
— — — —, evening, amel.: *Rhus.*
— — — — in morning: Arg., Par.
— — — — morning, irritation to, as from vapor of sulphur bath: *Chin.*
— — — — from tickling low in larynx: *Arn.*
— — **sleep**, when going to: Agar., Arn., Brom., Lach., Lyc., Nitr. ac.—10.
— — **smoking**, from: Clem., Coc. c., Hell., Ign., Lach., Petro., Nux v.—10.
— — — — in evening: Coloc.
— — **smothered** feeling, night Asaf.
— — **speaking**, from, morning: Sum.

**HACKING cough, sternum,** from tickling at lowest part of: Verat.
— — **stomach,** from crawling about pit of, extending to throat: Plat.
— — **stools,** excited by frequent: Bufo.
— — **sulphur** baths, irritation to, morning, after rising, as from: *Chin.*
— — **supper,** after: Natr. ars.
— — on **swallowing**: Kali hypermang.
— — **swallow,** effort to, immediately after: Cina.[7]
— — **teasing** sensation beneath right fourth and fifth ribs: Natr. ars.
— — **thickening** of mucous membrane of larynx, sensation of; with stringy, yellow expectoration: Ery. aqu.
— — **throat,** from burning in: Kali b.
— — — — crawling in: Colch., Euph., Lach., Prun. sp.
— — — — dryness in: Plan.
— — — — dryness in, afternoon: SANG.
— — — — dryness in, morning: Ant. t., Mang.
— — — — irritation in: Thuja, Tromb.
— — — — irritation in, much increased from heat and cold air: Hydras.[7]
— — — — itching in, morning: Ant. t., *Con.*
— — — — mucus in: Seneg.
— — — — rawness in: Kali b., Kali iod., Stront.
— — — — roughness in: Ang., Rhus.
— — — — scraping in: Laur., Ol. an., Prun. sp., Sin. n.

**HACKING cough, throat,** from scraping in, evening: Tereb.
— — — — scratching in: Sil.
— — — — stitches in: Chenop., Cist.—10.
— — — — — — with hoarseness: Carb. veg.[10]
— — — — tickling in: Colocn., Mur. ac., *Phos.*, Plan.
— — — — tickling in, evening: Amm. m., *Sang.*
— — — — tickling in, forenoon: *Amm. m.*
— — — — tickling low in: Dios., Tarax.
— — — — tickling in, morning: Sum.
— — — — tickling at top of, noon: Naja.
— — — — on touching the: LACH.
— — from putting **tongue** out: Lyc.[10]
— — on touching **swollen tonsils**: Phos.
— — **trachea,** from irritation in: Gels.
— — — — mucus in: Gels.
— — — — rawness in: Laur.
— — — — sensation of something in: Sin. n.
— — — — tickling in: Carb. ac.
— — — — tickling in, evening, 7.15 P. M., on entering warm room: Com.
— — — — tickling low in, morning, after rising: *Arn.*
— — **violent,** with expectoration of a mouldy taste and smell: BORAX.
— — on **waking**: Phos.
— — — — in morning, agg.: Sil.
— — **walking,** in open air: Ang.
— — — fast, agg.: Seneg.

**HÆMORRHOIDS, pain** in, during cough: Kali c., Lach.[10]
- **stitches** in, on coughing: *Ign.*, Lach.—17.

**HAIR**, sensation of, in trachea, excites cough: Sil.

**HANDS cold**, with heat in face and head, with coughing on going to sleep: *Sulph.*[6]

— must **hold** chest with both, when coughing: ARN.,[10] Bor.,[6] Bry., Cimic.,[7] Dros.,[6] Eupat. per., Kreos., Merc., Natr. s., *Sep.*,[10]—7.

— **laying** on pit of stomach amel. cough: *Croc.*,[1] Dros.[6]

— **sweat** on, during cough: Ant. t.

— **uncovering** even a, excites cough: HEPAR, *Rhus*,[7] Sil.—2.

**HARD** cough: Asc. t., Aur. s., Bell., Calc., Cann. i., Caps.,[5] Cupr., Eupion, Gymn.,[5] Kali b., Linu., Lyc.,[5] Naja, Nux v.,[5] Rhus, *Sep.*, Ziz.—1.

— — **evening**: Apoc. c., *Puls.*

— — **larynx**, from scratching in, amel. by discharge of mucus: Alumn.

— — **night**: Apoc. c.

— — **palate**, from tickling in, after lying down: Carbon. s.

— — while **smoking**: All. s., Nux v.[10]

— — spells of, not ceasing till masses of offensive sputa are raised: Carbo v., Ranc.

— — **throat**, from tickling in, evening: Gymn.

— — **trachea**, from tickling in: *Psor.*

**HARASSING.** See Teasing Cough.

**HARSH**, hard cough: Eupat. per.[5]

— See Rough Cough.

**HAWKING** excites cough: Amm. m.

— short cough: Eugen.

— with cough: Coc. c., Lach.—5.

**HEAD, aching** (includes pain, etc.) in, on coughing: Acon.,[5] Alum., Ambr., Anac., Ang.,[12] Apis,[20] *Arn.*, Ars., Aur., Bad.,[2] BELL., BRY., *Calc.*, Cact.,[12] *Caps.*,

**HEAD, aching**—(*Continued*). Carb. v., Caust., Chin.,[5] Chel., Con., Ferr., Hepar, Ign.,[2] Ipec., Iris v., Kali b., *Kali c.*, Lach., Led.,[5] Lyc., Mag. s., Mang.,[12] *Merc.*, NATR. M., Nitrum, Nitr. ac., NUX V., Œna.,[1] Ol. m.,[20] Phos., Ph. ac., *Puls.*, Rhus, Rumex,[1] *Sabad.*, Sang.,[5] Sars.,[5] Sep., Spig., Spong.,[2] *Squil.*, SULPH., *Stann.*, Tarent.[1]—11.

— **aching** in, agg. by coughing: Iris, Sulph., Tril.—1.

— — after coughing: *Stann.*

— — alternating with cough: Lach.

— — in and stoppage of breath during cough: Squil.[6]

— — behind eyes, worse on turning the head, with the cough: Bad.[2]

— — with dry cough, coryza and stitches in the throat: Nitr. ac.[10]

— — in forehead, with cough: Spong.[2]

— — in occiput, on coughing: Alum., Sulph.

— — in occiput, soreness in chest, tearing pain in temples and vertex, from coughing: Alum.[2]

— — pressure from within outward, with cough: Kreos.[2]

— — stupid feeling in, with noises in ears, on coughing: Ph. ac.[20]

— — in temples, from cough: Alum., Bry., Rhus, Sulph.—2.

— — when sneezing and coughing, disappearing on compressing the head: *Natr. m.*[6]

— **beating** pain in, after cough, and in pit of stomach: Ipec.[6]

— **beatings** in, as of hammers, bursting pain in forehead, with cough: Natr. m.[7]

— **blood** congestion or pressure of, to, on coughing: Acon., Ambr., Anac., Bell., Calc., Carb. v., Caust., Cham., Chin., Dulc., Hyos., Iod., Kali c., Lach., Laur., Lyc., Mag. c., Mag. m., Merc.,

**HEAD**, **blood**—(*Continued*).
Mosch., Nitr. ac., Nux v., Phos., Rhus, Samb., Seneg., Sep., Sil., Spong., Stram., Sulph.—2.
— **blood**, congestion of, to, from chest, with throbbing on coughing: Sulph.—2.
— **blows**: See Shocks.
— **brain**, sensation as of looseness of the, with cough: Acon., Sep., Sul. ac.[2]
— — stitches in left side, deep in, on coughing: Bry.[6]
— — tearing at a little spot in, on coughing: Sep.[6]
— **bruised** feeling in, on coughing: Mur. ac., Sulph.,[1] Verat.—2.
— — feeling in occiput, on coughing: Tarent.
— **burning**: See Heat.
— **burst**, violent racking cough, as if head and chest would, worse at night: *Merc.*[7]
— **bursting**, sensation in, when coughing: Bell., Cact.,[12] *Caps.*,[12] Chin., Hydras.,[20] Kali b.,[5] Merc., *Natr. m.*, Nux v., Phos., Ph. ac., Puls., Sep., Sil., Staph., Sulph.—2.
— — sensation in, agg. by cough: Bell., Hydras., Sep.[7]—1.
— — sensation in forehead, when coughing: Natr. m.,[7] Staph.[2]
— — sensation in, morning, 8 A. M., on coughing: Dios.
— — — — morning, 11 A. M., on coughing: Lac. ac.
— **cold** feeling, on one side of head, accompanies cough: Calc.[2]
— **compressive** pain, with cough: Arn., Verb.[2]
— — — in forehead and temples, during cough: Verb.[2]
— **concussion** of the brain, from cough: Apis,[7] Led., Rhus, Sul. ac.—2.
— **constriction** of, on coughing: Iris v.,[17] Petro.[1]

**HEAD**, **creeping** sensation in, with cough: Cupr.[2]
— **cutting** pain in, on coughing: Bell.[20]
— **darting** pains in parietal bones, on coughing: Mang.[5]
— **digging** pains in temples with the cough: Kali b.[5]
— **exertion** of mind, agg. cough: Arn., Ign., Nux v.—2.
— **fly to pieces**, feels as if it would, from the cough: Bry., Calc.,[6] Caps., Kali b., Merc., Natr. m., Nux v., Phos., Ph. ac., Sep., Sulph.—5.
— — — — feels as if head and chest would, from violent racking cough, every other evening, when on the point of going to sleep; cough is followed by violent stretching: Merc.[6]
— — — — See also under Bursting.
— **forehead**, aching in, with cough: *Asc. t.*, Brom., Chel., Spong.[2]—1.
— — bursting sensation in, with cough: Staph.
— — constriction of, when coughing: Iris v.[17]
— — pains in, from coughing: Acon., Arn., Hepar, Hyos., Kreos., Mosch., Natr. m., Phos., Ruta, Seneg., Spong., Staph., Sulph. Verb.—2.
— — piercing pains in, on coughing: Hyos.[2]
— — pain in, and swimming in head, after cough, almost causing one to fall: Kali b.[6]
— — pain in, relieved by cough: Arg. mur.[20]
— — pressure of blood and throbbing in, with cough: Spong.[2]
— — stitches in, with cough: Hyos.,[2] Sulph.[6]
— — stitches through, when coughing, is obliged to hold it with her hands: Sulph.[6]

**HEAD, fullness** in occiput, with heat and fluent acrid coryza, lacrymation, and cough: Cepa.[2]
— **hammering** in, from cough: Natr. m.[2]
— **heat** of, during cough: Amm. c., Ant. t., *Ars.*,[1] Carb. v., Ipec., *Sulph.*[1]—5.
— — — and rest of body cool: Arn.[5]
— **heat** of, and rest of body cool, with cough. Arn.[5]
— — in face and head with cold hands, with cough when going to sleep: *Sulph.*[6]
— **heaviness** and dullness in, with cough: Euphr.[2]
— **hold**, splitting headache, child holds its head, whooping cough, with vomiting, worse mornings: Nux v.[7]
— **hold**, stitches through the forehead, when coughing, is obliged to hold it with hands: Sulph.[6]
— **hold**, violent cough, must sit erect and hold head with both hands: Nicc.[14]
— **jerks** in. See Shocks.
— **jerking**, tearing in temples, with cough: Puls.[2]
— **lightness** in, with chronic, loud cough from stuffing at epigastrium, chiefly in morning; has then a fit of coughing, and expectoration of tough mucus: Kali b.[6]
— **looseness** of brain, sensation as of, with cough: Acon., Carb. an., Sep., Sul. ac.—2.
— **mixed** reverberation in.
— **motion** in, on coughing: Bry.[6]
— **occiput**, aching in, on coughing: Alum.,[2] Sulph.
— — bruised feeling in, on coughing: Tarent.[1]
— — pains in, on coughing: Anac., Carb. an., Ferr., Mag. c., Mosch., Sep.—2.

**HEAD, occiput**, ulcerative pain in, when coughing: Sep.,[2] Sulph.[6]
— **oppression** of chest and, with vertigo and cough: *Brom.*[6]
— **pains** (undefined) in forehead with or from cough: Acon., Hepar, Hyos., Kreos., Mosch., Natr. m., Phos., Seneg., Spong., Staph., Verat.—2.
— — See Aching.
— **pain** in forehead and swimming in the: Kali b.[6]
— **pain** coming over from nape of neck, on coughing: Carb. v., Sil. —2.
— **pain** in occiput, from cough: Anac., Carb. an., Ferr., Mag. c., Merc.,[11] Mosch., Sulph.,[11] Sep. —2.
— **pain**, tearing in, from violent dry cough: cough followed by violent pulsation of heart; anxiety and pressure in chest, worse sitting: *Cupr.*
— **pain** in temples with cough: Ambr., Caust., Cina, Kali c., Kreos., Lyc., Puls., Rhus, Verb. —2.
— **pain** in vertex on coughing: Con., Cupr., Sabad., Squil.—2.
— — violent, in centre of, from coughing or sneezing: Sulph.[6]
— **parietal** bones, darting in, from cough: Mang.[7]
— — — stitches in, when coughing: Sulph.[6]
— **perspiration** on, with cough: Ant. t., Calc., Ipec.,[12] Sil., Tarent.[1]—5.
— — sour on, with cough: Merc., Sil.—5.
— **pressing** from within outward in the forehead, with cough: Acon., Hepar, Kreos.—2.
— **pressing** or pain in, agg. by coughing: Sep.[7]

**HEAD, pressure** of blood to. See Blood, pressure of.
— — in forehead and throbbing, with cough: Spong.[2]
— **pressure** in, when coughing: Alum., Bry., Chel., Con., Nitr. ac.,[1] Phos., Ruta, Sars.—5.
— **pulsation** in, during cough: Lyc.
— **racking** of, occiput, abdomen, and chest from short turns of cough: Lact.[6]
— **roaring** in, with cough: Caust., Hepar, Mag. m.—2.
— **shaking** in, during cough: Chin., Lact., Mag. s.
— **shattering** in, during cough: Calc., Lact., *Rhus.*
— **shocks** (or jerks) in, from cough: Ars., Calc., Ipec., Lach.,[12] Lyc., Mang.,[12] Natr. m., Rhus, Seneg.,[2] Spig.,[12] Sul. ac.—5.
— **shooting** in, when coughing: Bry., Calc., Carb. v., Con.—1.
— **shooting** pain in, on coughing; stitches extending upward are renewed when coughing or moving head, only relieved when resting head on painful side: Arn.[6]
— **sensibility** in vertex, with cough: Squil.[2]
— **side** of, aching in left, agg. by cough: Apis, Dir.
— — —, boring in, agg. by cough: Aur.
— — —, darting pain in, from cough: Mang.[7]
— — —, grinding in, agg. by cough: Aur.
— — —, pain in temple, from cough: Mang. (r.).
— — —, pulsation in, agg. by cough: Aur., Dir. (l.).
— — —, shooting in, from cough: Mang.
— — — sticking pain through to arm, agg. by cough: Cimex (r.).

**HEAD, side** of, stitches in occipital and parietal bones, on coughing: Sulph.
— **soreness** of, when coughing: Spig.,[1] Zinc.[2]
— **spasmodic** pain in, on coughing: Ery., Mar.[20]
— **split**, pain with cough, as if would split at forehead, with stitches in throat: Hepar.[6]
— **splitting** pain, child holds its head, whooping cough with vomiting, worse mornings: Nux v.[7]
— **stitches** in, when coughing: Alum., Anac., Arn., Bry., Calc., *Carb. v.*, Caust., Chel., Cina, Con., Hyos., Kali c., Mez.,[5] Nitr. ac., Phos., Ph. ac., Ruta,[5] Sabad., Sulph., Sul. ac., Verb., Zinc.[1] —2.
— **stitches** over eye, with cough: Phos., Ph. ac.—2.
— —. in forehead during cough: Hyos.,[2] Mez., Sulph.—6.
— — through forehead, when coughing, is obliged to hold head with her hands: Sulph.[6]
— — in right frontal eminence and scraping at lower part of sternum, with cough: Mez.[6]
— — in left hemisphere of brain, on coughing: Bry.[6]
— — in temples, with cough: Caust., Cina, Kali c.—2.
— — in vertex, during cough: Con., Sabad.—2.
— — in vertex, from dry cough, with perspiration and water in eyes, vomiting and pain in stomach: Sabad.[10]
— **stretching** back, excites cough: Lyc.[2]
— **stunning** pain in, with cough: Aeth.
— **stupefying** pain in, cough awakens him at 3 A. M. with: Nitrum.[7]

**HEAD, stupefying** pain in, with cough: Led.,[2] Nitrum.[7]

— **sweat** on. See Perspiration.

— **tearing** in, with cough: Alum., Calc., Cupr., Acet.,[6] Sep.—5.

— **tearing** pain in, agg. by coughing: Arn.

— **tearing** asunder, pain as if, with cough: Mur. ac., Puls., Verat. —2.

— — See also would fly to pieces.

— **tearing** pain in, from frequent, violent, dry cough, followed by violent pulsation of heart; anxiety and pressure in chest: *Cupr. acet.*

— **tearing** pain in small spot in brain, with cough: Sep.[6]

— **tearing** pain in temple, from cough: Alum., Puls.[12]

— **temple**, pain in, with cough: Ambr., Caust., Cina, Kali c., Kreos., Lyc., Puls., Rhus, Verb. —2.

— **temple**, pain in, and to top of head, from cough: Alum.[7]

— — sensation as if right temple would burst, when coughing: Cina.[6]

— — stitches in, from cough: Caust., Cina, Kali c.—2.

— — tearing in, from cough: Alum., Puls.—2.

— — tension in, with cough: Verb.[2]

— **tension** in, with cough: Mag. c., Merc., Mosch., Verb.—2.

— — — forehead: Mosch., Verb. —2.

— — — occiput: Mag. c., Mosch. —2.

— — — temples: Verb.[2]

— **tensive** pain in, on coughing and loud speaking: Spig.[20]

— **throbbing** in, with cough: Ferr., Ipec., Kali c., Led., Natr. m., Nitr. ac., Phos., Seneg., Sep., Sil., Spong., Sulph.—2.

**HEAD, throbbing** in forehead, with cough: Phos., Spong.—2.

— — in occiput, with cough: Sep.[2]

— **top** of, pain in temple and, from cough: Alum.[7]

— **turning**, excites cough: Spong.[5]

— **twitching** in, on coughing: Puls.[2]

— **ulcerative** pain in occiput, with cough: Sep.,[2] Sulph.[6]

— **vibration** in, during cough: Hepar.[1]

— **awakens** at 3 A. M., with cough and violent stupefying headache: Nitrum.[7]

— **weakness** in, after cough: Hepar.[1]

**HEARING** impaired by coughing: *Chel.*, Dulc.,[7] Seneg.[1]—11.

**HEART, affections** of, with cough: Ars., Lach., Laur., Naja, Mosch. —5.

— *and arteries*, dry cough, after midnight, causing violent beating of: Calc.[6]

— **burn**, with, from cough: Carbn,s.

— **constriction** of, with cough: Cact.[5]

— **sensation of obstruction** in region of, suddenly, as if breath would be arrested; attacks are frequent, occur even at night during sleep, and excite a dry cough, which is relieved by expect.: Guaj.[6]

— **sensation** of **obstruction** in region of, preceding the cough: Guaj.[6]

— **pain** about, during cough: Tarent.

— **palpitation**, with cough: Arn., Calc., Kali b., Nitrum, Puls., Spong., Stam.—5.

— **palpitation** of, agg. by cough: Ib.

**HOARSE cough**—(*Continued*). an., Carbo. v., Caust.,[1] Chin., Cina, Dros., Eupat., Hal.,[1] Hepar, Kali bi., Kali iod.,[1] Lach., Laur., Merc., Naja,[1] Natr. m.,[1] *Rhus*,[1] Samb., *Sil.*,[1] Spong., STANN., Sul. ac., Verat., Verb.—5.

— **choking** cough, evening; Cina.

— **croupy** cough: Ant. t., Bell., *Brom.*, Lach.—5.

— **hacking** cough: Acon.[5]

— dry, rough, and barking cough (also a panting cough) worse on deep insp.: Dulc.

— mostly morning and evening, not at night: Caust.

— filling-up sensation in throat: Apis.

— racking cough, day and night, with profuse purulent expectoration; soreness in larynx: Ars. iod.

**HOARSENESS**, with cough: Acon., Amm. c.,[16] *Ant. t.*,[11] Arg. n.,[11] Asaf., Bad.,[2] *Bell.*,[11] Bry., Brom.,[2] Calc., Caps.,[11] Carb. an., CARB. V., Caust.,[2] CHAM.,[11] Chin., Cina, Cupr.,[2] Dig.,[2] DROS.,[11] *Dulc.*,[16] Ferr.,[2] Hepar, Iod., Kali b., Kali c.,[2] Kali hyd., Lach., Laur., Lyc.,[12] Mag m.,[16] Mang., MERC.,[11] Mez.,[2] Mur. ac.,[2] Natr. c., Natr. m.,[16] Nitr. ac., Nitrum,[16] *Nux v.*,[11] Phos., Ph. ac., *Puls.*,[11] *Rhus*,[11] Rumex, *Samb.*, Seneg.,[16] Sep., Sil.,[12] Spong., Stann., Staph.,[2] Sulph.,[11] Sul. ac.,[2] Thuja,[16] Verat., Verb.,[2] Zinc.[2] —5.

— **afternoon**, during cough: Gamb.

— **amel.** by coughing: Stann.

— with **desire** to cough: Alum., Chinin., Dros., Laur., Sul. ac.—16.

— with **dry** cough: Con., Sep., Sil. —16.

**HOARSENESS**, with **dry** cough, dryness in throat and coryza of clear water: Sulph.[6]

— dry cough, from 5 to 6 paroxysms with sore sensation in a streak down along the trachea, where it pains at every paroxysm of cough, and almost prevents breathing: CAUST.

— **morning**, with cough: Carb. an., Dig.—2.

— day and night; cough, with soreness and dryness of throat and chest; expectoration yellow; more in morning: Calc. ph.

— **nightly**, with dry cough and blood-streaked saliva: Arg. n.[6]

— with **tedious** cough: Bar., Calc., Caust., Iod., Mang., Phos. —16.

— sudden, violent, with dry cough and pressure on chest during rough weather: Mag. m.[6]

— with violent dry cough, after measles: Camph.[7]

**HOLD**, cough obliges him to hold himself inwardly: Coff.

— See also under Chest, Head, etc.

**HOLLOW** cough: Acon.,[5] Ant. t.,[5] BELL., Carb. v., *Caust.*, Chel., Cina,[5] Dig., Euphr.,[5] Hepar,[5] Ipec., Jatro.,[1] Kali iod.,[1] Lach., Lact.,[1] Kreos., Led., Mag. c.,[1] Merc. c.,[1] Natr. c.,[1] Nux v., Op., *Phos.*, Samb., Sil., Spig., Spong., Stann., Staph., VERAT., Verb.—2.

— — in **bed**, in morning: *Phos.*

— — in **day**: Spong.

— —, **deep**, toward noon, with discharge of pure blood: Sil.[6]

— — **dry** cough, from five to six paroxysms, with sore sensation in a streak down along the trachea, where it pains on every paroxysm of cough and almost prevents breathing: CAUST.

— — in evening: Verat.

**HEART**, **pulsation** of, frequent violent dry cough, with tearing pain in head; cough followed by violent pulsation of heart; anxiety and pressure in chest, worse sitting: *Cupr acet.*

— **shocks** of, for two hours after dinner, obliging her to cough frequently and causing flushing of the face: Phos.[6]

— **shooting** in region of, agg. by cough, hiccough, sneezing: Agar.

— **stitches** in region of, when coughing: Mez.

— **tearing** in region of, with cough: Elaps.[5]

— **thumping** of, after dinner, excites cough: Phos.

**HEAT** after the cough: Bell.

— **agg.** or excited by coughing: Ambr., Ant. t., *Arn.*, *Ars.*, Bell., Carb. v., Hepar, Hyos., Iod., Ipec., Led., *Lyc.*, Mag. m., Natr. c., Nitrum, Nux v., Phos., Puls., Sabad, Squil., Sulph.—3.

— of **body** excites dry cough: Acon.

— in **bronchi**, excites cough: Eth.

— with cough, with expectoration: Alum., Anac., Arg., ARS., Bell., Brom., *Bry.*, CALC., Carb. v., Cic., *Chin.*, Dig., Dros., Dulc., Ferr., Iod., KALI C., Kreos., Lyc., PHOS., Ph. ac., PULS., Ruta, Seneg., Sep., *Sil.*, Spong., Stann., Staph., Sulph., Thuja.—3.

— with cough, without expectoration: ACON., Ang., Ant. cr., *Apis*, *Arn*, Ars., Bell., Brom., *Bry.*, Calc., Carb. v., Caust., Cham., Cina, Chin., Coff., CON., Cupr., Dros., Hepar, *Hyos.*, Ign., IPEC., *Kali c.*, Lach., Lyc., NATR. M., Nitr. ac., Nux m, NUX V., Op., Petro., *Phos.*, Plat., Puls., *Rhus*, SABAD., Samb., Sep., Spig., Spong., Squil., Staph., *Sulph.*, Sul. ac., Verat., Verb.—3.

**HEATED**, cough, after being: Acon., Bry., Caust.,[5] Kali c., Mag. c.,[5] Nux m.,[5] Rhus, Zinc.—2.

— on **becoming**, agg. of cough: Acon.,[5] Ant. cr., Bry.,[5] Caust.,[5] Dig., Iod., Kali c., Nux m.,[5] Nux v.,[5] Sil., Thuja.—2.

— See also under Warm, Warmth.

**HEAVINESS** in chest, from dry cough: Sulph.

— **extending** into chest from throat, causes cough: Iod.

— of **chest**, with cough: Amm. c., Calad., Kali b., Nitrum, Sep.,[5] Zinc.—5.

— and dullness in **head**, with cough: Euphr.[2]

**HERNIA**, pains as if, would set in on coughing: Cocc., Natr. m., Nux v., Sil., Squil., Sulph., *Verat.*—11.

— when walking and standing pain in lumbar region, as if hernia would protrude; dares neither cough nor inhale: Sul. ac.[6]

— See also under Abdomen, protrude.

**HICCOUGH** during cough: Ang.,[5] Tabac.[1].

— and after cough: Tabac.[10]

— and sobbing, with cough: Amm. m.

**HIP**, **jerking** or twitching in, seems to excite cough: Ars.

— pain in, with cough: Bell., *Caust.*, Rhus, Sulph.[11]—5.

— **stitches** in chest, lumbar region, hip, uterus, with cough; pain in sternum, with tightness of chest and rattling of mucus in chest: Bell.[10].

— **stitching** pain over left, extending to small of back, when coughing: Sulph.[6]

**HOARSE cough**: ACON., ALL. C.,[1] Aloe,[1] Ambr., Asaf.,[1] BELL., Brom., Calad.,[1] Calc.,[1] Carbo

**HOLLOW** cough from tickling in **chest**, during rest: Euph.

— — after **lying down**, evening: Lact.

— — **morning** and night, agg.: Caust.

— — **morning** in bed: *Phos.*

— — **night**: *Phos.*, Spong.

— — —, especially at night and in the morning, with tightly adherent mucus in chest, in which there is a sticking pain, very sore and ulcerated, with and without coughing, with stopped coryza and stoppage of nose: *Caust.*

— — toward **noon**: Sil.

— — after **rising** in morning: Cina.

— — **stooping** agg.: Spig.

— —, **sitting** up amel.: Natr. c.

— — on **waking** in morning: Ign.

**HOT** room, unable to speak from excessive coughing in a: Coc. c.

— See also under Room.

**HUNGER**, violent, with cough and coryza: Sul. ac.[6]

**HYPOCHONDRIUM**. See under Abdomen.

**HYPOGASTRIUM**. See under Abdomen.

**HYSTERICAL** attack of cough followed by crying, night: Formica.

**ILL-HUMOR** before the cough: Asaf., Bell.

**IMPULSE**. See Irritation.

**INABILITY to cough**: Ant. t., Sulph.

— — — from pain: Natr. slfc.

— — — — — in side: Ox. ac.

— — — — — amel. by pressure of hand on pit of stomach: *Dros.*

**INCESSANT.** See Constant.

**INCLINATION**. See Irritation.

**INDOORS**, slight cough: Coca.

— See also under Room.

**INGUINAL** region, pain in on coughing: Borax.[11]

— —, **pain** in, as if hernia would protrude, on coughing: Petro.

— — See also under Hernia, and Abdomen.

— — **stitches** in, during cough: Lach., Thuja. (r.)

— — **tearing** in, as from hernia, agg. on coughing: Tarent.

**INHALE,** inclination to cough so sudden and violent can scarcely inhale at all: Sepia.[7]

— See Breathing.

**INSPIRATION** (inspiring, inhaling) agg. or excites cough: Acon., Asaf.,[1] Asar., Benz. ac.,[1] Calc.,[1] Cina, Con., Coral., Croc., Graph., *Hepar*, Ipec., Kali b., Mag. m.,[1] Meny., Meph.,[12] Merc. p. iod., Olean., Op., Puls., Squil., Sticta, Verb.—12.

— **agg.** a dry cough, evening: Dig.

— — short cough; also a dry night cough: Natr. ars.

— See also under Breathing.

— **choking** cough from: Cina.

— **crowing**, violent spasmodic cough, commencing with gasping for breath, and continuing with repeated crowing inspirations till face becomes black or purple and patient exhausted; worse at night and after a meal: *Coral.*[2]

— **deep**, cough from: Am. m.,[16] Brom., China,[16] Cina,[16] Con., Coral., Cupr., Graph., *Hepar*, Lyc.,[16] Natr. ars., Natr. m.,[16] Serp. Squil.[16]—1.

— **deep**, agg. hacking cough: Natr. ars.

— —, amel. cough: Verb.[12]

— —, amel. hacking cough: Osm.

— on **deep**, it seems as though icy-cold air were streaming through the air-passages, with some provocation to cough, and much dif-

**INSPIRATION**—(*Continued*). ficult hawking of bronchial mucus, in the morning: *Coral.*

— **deep**, causes strangling cough, red face, watery eyes, and straining all over: Graph.[7]

— **deep**, after lying down, evening, cough: Ipec.

— — — — — morning, cough: Ipec.

— — racking cough from: Con.

— — short cough from: Con.

— — tight cough from: Ziz.

— **dry** cough on: Hepar.

— **evening**, agg. dry cough: *Dig.*

— on **forced** through nose, cough: Sap.

— **irritation** to cough on: Physos., *Puls.*

**INSPIRING cool air** agg. cough: *All c.*, Cist., Cupr., Phos.,[10] *Rumex*,[2] Staph.,[2] Vitex.—5.

— — — See also under Air.

— **deeply**. See Breathing.

— **every effort** excites cough: Meny., Sticta.—2.

**INTERMITTENT-FEVER**, dry hacking cough in spells before the attack of: Eupat. per.[7]

— — See Fever.

**INTERMITTING** cough, 6 A. M. amel. drinking cold water: *Coc. c.*

**INTERRUPTED** cough: Coff., Sulp. ac., Thuja.

— — after dinner: Agar.

— — from **infiltration** of lower chest: Kreos.

— — from smoking, evening: Thuja.

**INVOLUNTARY** cough: Aml. n.

— half, rough cough, from constant roughness and crawling in throat: *Carbo v.*

**IRRITABILITY** with cough: Cham., Cina.—5.

**IRRITABLE cough**: Cocc., Coff., Clem.,[1] Cod.,[1] Ign., Kali c.,[1] Lach., Laur., Ozone, Mar., Par., Ph-ac.,[1] Phyto.,[1] Plan.[1]—5.

— — **back**, lying on or right side agg.: Phos.

— — **evening** tickling in trachea, after lying down: Marum.

— — **lying** on back or right side agg.: Phos.

— — **lying** down, after, evening, from tickling in trachea: Marum.

— — **morning**, after rising: Arg.

— — **night**, agg.: *Cod.*

— — from **scratching** in throat: *Phos.*

— — on **swallowing**: Kali hypermang.

— — — **tickling** in upper trachea: Marum.

**IRRITATION** (desire, inclination, etc.) **to cough**: Acon., *Agar.*, All. s., Aloe., Am. bro., Am. m., Aml. n., Ant. t., Aqu. p., Asp., Baryt., Cai., Carb. v., Carb. ac., Carbn. s., *Caus.*, *Cham.*, Chin. s., Chlo., Clem., Coc. c., Coll., Con., Crot. t., Dios., Hyos., Iod., Kali b., Kali iod., Lobel., Lyc., Mag. s., Merc. i. r., Mez., Mosch., Mur. ac., Natr ars., Natr. slfc., Nux v., Osm., Ox. ac., Phos., Ph. ac., Psor., *Pul. n.*, Raph., Sulph., Sul. ac.

— **to cough**, afternoon: Bapt., Sulph.

— — — — 7.30 P. M., speaking: Cimic.

— — — **back**, lying on: Natr. m.

— — — during **catarrh**: Ant. t.

— — — during **coryza**: Sepia.

— — — agg. by **coughing**: Ign.,[1] Marum.,[12] Raph.

— **to cough**, afternoon: Bapt.

— — — **afternoon**, 7.30 P. M., agg. on speaking: Cimic.

**IRRITATION to cough, afternoon**, agg. in: Sulph.
— — — **back**, lying on: Natr. m.
— — — in **bronchi**: Chlor.
— — — **catarrh**, during: Ant. t.
— — — **chest**, from burning in: Coc. c.
— — — — from tickling contraction in: Alco.
— — — **coryza**, during: Sepia.
— — — **coughing** agg.: IGN., Marum,[12] Raph.
— — — **day**: Agar.
— — — — smoking: Agar.
— — — after **dinner**: Sulph.
— — — — **eating**: Sulph.
— — — while eating felt in larynx *Rumex.*
— — — **evening**: Chel., Dios.
— — — — lying down: IGN., Mez., Sulph.
— — — —, toward, in larynx: Merc. c.
— — — — 2 P. M., in larynx and trachea: Coca.
— — — — 7.30 P. M., tickling in larynx: Cimic.
— — — — 8 P. M., pain in right lung: Dios.
— — — — 9 P. M., tickling in throat: Dios.
— — — — irritation in throat: Dios.
— — — **fauces**, from tickling in: Aloe.
— — — **increases** the more one coughs: IGN., Marum, Raph.,[1] Squil.[6]—12.
— — — on **inspiration**: Physos., *Puls.*
— — — — deep inspiration, morning: Coral.
— — — in **larynx** and trachea, 2 P. M.: Coca.
— — — — while eating: *Rumex.*
— — — — — after eating: Staph.
— — — — — constriction and irritation: Naja.

**IRRITATION to cough** in **larynx** toward evening: Merc. c.
— — — — — from pain: *Iodium.*
— — — — — from scraping: *Con.*
— — — — — from stitches: Naja.
— — — — — from tickling: *Brom.*, Cimic.
— — — — — from tickling, 7 P. M.: Cimic.
— — — **lung**, from pain in right, 8 P. M.: Dios.
— — — **lying** on back: Natr. m.
— — — — down, evening: Ign., Mez., Sulph.
— — — about **midnight**: Coff.
— — — before midnight: STANN.
— — — **morning**: Dios., Meny., Ol. an., Thuja.
— — — — in bed, agg.: *Caust.*
— — — — on deep inspiration: *Cori v.*
— — — — after rising: Alumn.
— — — **night**: Com., Ferr.
— — — **palate** from burning in: Acon. f.
— — — — — — up to arch of: Dig.
— — — from **reading** aloud: Nitr. ac.
— — — **respiration**, from deep: Cina, *Lyc.*
— — — — on deep, morning: *Cori. r.*
— — — **rising**, after, in morning: Alumn.
— — — — amel.: Ferr.
— — — **smoking**, agg., day: Agar.
— — — from **speaking**, 7 P. M.: Cimic.
— — — **sternum**, tickling, under extending to back: August.
— — — **sulphur** fumes, as from: Carb. v., Lyc.
— — — **swallowing**, agg.: Eug.
— — — — empty: Lyc., Natr. m.
— — — in **throat**: *Amm. br.*, Tabac.

**IRRITATION to cough** deep in throat: Ambra, Asp., Dios., Squil.
— — — **throat**, from draft of air: Plan.
— — — — burning in: Acon. f.
— — — — dryness in: Atro.
— — — — irritation in, 10 P. M.: Dios.
— — — — from sensation as from fumes of burning match: Aml. n.
— — — — sticking griping; Merc. ac.
— — — — sticking: Phos.
— — — — on stretching it: Lyc.
— — — — from tickling in: Asp., Natr. c.
— — — — in pit of throat: Ph. ac.
— — — from tickling in throat, 9 P. M.: Dios.
— — — **tongue**, from burning on: Acon.
— — — in **trachea**: Stann.
— — — — — and larynx, 2 P. M.: Coca.
— — — on **waking**, night: Thuja.
**ISCHIATIC** pain on coughing: Ars., Bell., Caust., Kali b., Sulph.—12.
**JAW**, pain in when coughing, not on touch: Amm. c.
**KNEE, pain** in, or leg, on coughing: Caps.[12]
— **shooting** in when coughing: Nitr. ac.
**LABOR**, dry cough following difficult labor or abortion, with backache and sweat: Kali c.
**LABORED** cough: Agar.
**LACHRYMATION**, when coughing: Acon., Agar.,[1] All. c., Brom., Calc., Calc. ph., Carbo v., Chel.,[5] Cina, Eupat. per.,[12] EUPHR., Hepar, Ipec., Kali c., Kreos., Merc., Natr. m., Op., Phell., Puls., Rhus, Ruta,[6] Sabad., Sil., Squil.,[5] Staph., Sulph.—2.
**LACTATION**, cough and bloody expectoration during: Ferr. acet.[6]
**LAMENTING**, cough from, in children: *Arn.*
— see Crying.
**LARYNGEAL** cough, compels patient to grasp the larynx: *All. c.*[7]
— cough: Carbn. s.
— —, as from foreign body: Bell.
— —, originating above larynx: Calad.
— —, as from sulphur fumes in upper part of larynx: *Ars.*
**LARYNX, air**, inspiring in, causes dry cough: Ment. pip.
— — — — laryngeal cough: Meng.
— — open, stitches and tickling in, while walking in open air, exciting cough: Ox. ac.
— **back part** of, irritation of, evening in bed, excites paroxysmal cough: Cocc.
— — —, tickling at, causes dry cough: Anth. n.
— — —, — — — paroxysmal cough: Anth. n.
— **balls** of soap-like mucus accumulates in, causing cough: Arg. n.[6]
— **burning** in, causing cough: Acon.,[2] Aph., Bell., Mag. s.,[12] Phos., Seneg.[5]—1.
— — in, with cough: Ars., Cham., Iod., Mag. m., Phos.—2.
— — from pit of stomach to, with cough: Mag. s.[5]
— — tickling in, exciting cough: Aph.,[1] Spong.[5]
— **clothes**, cough from pressure of, on: LACH.[5]
— **cold** sensation in, on inspiration: Brom.[2]

**LARYNX, constant** desire to clear the throat and: Crot. t.
— **constriction** of, excites cough: Bell., Carbn. s., Cupr.,[2] Mang., Naja, Puls., Stram.,[5] Sulph.—1.
— — in, causing spasmodic cough: Coffea.
— **constrictive** sensation in, especially after eating, causing cough: *Puls.*
— — tickling from upper part of, to lowest bronchi, causing cough: *Ipec.*
— **contraction** of, with cough: Dros., Ign., Stram., Verat.—2.
— — — when speaking: Dros.[2]
— **cramp** in, a kind of, as in whooping cough, during cough: Tereb.
— **crawling** (creeping) in, exciting cough: Ant. t.,[2] Carbo v., Caust., Con., *Dros.*, Kreos., Led., *Psor.*, Rhus, Sab., Sulph.
— — below, excites cough: *Kreas.*[5]
— — in, dry cough: Psor.
— — — hacking cough: *Dros.*, Psor.
— — — paroxysmal cough: *Psor.*
— **drink,** getting in, excites cough: Acet. ac., Acon.
— **drinking** water agg. racking cough from tickling in: *Opium.*
— **dry** spot in, causes cough: Con.
— **dryness** in, excites cough: Bell., Carbo an., Con., Dros., Laur., Mang.,[1] Puls.,[5] Seneg.,[6] Zig.—2.
— — — choking or suffocative cough 5 A. M., cannot speak on account of cramp in chest, red face and sweat: *Kali c.*
— — — on coughing: Bell., Polyg.
— — — tight cough: Ziz.
— — — with cough: Dros., Laur., Mag. m., Natr. m., Phos., Rhus, Sabad., Spong., Verb.—2.
— — roughness and constriction of, from dry cough brought on by reading and talking: Mang.[10]

**LARYNX, dust**, cough as from, in: Calc., Ph. ac., Sulph.—5.
— **feather**, tickling as from a, cough from: Dros.,[5] Mez.[1]
— — — — —, evening, before sleep, an overpowering cough from: *Lyc.*
— **flapping** sensation above: Lach.[2]
— **flesh** hanging in larynx, sensation as from a piece of. Phos.
— **fluid** secretion of in, cough from: *Lach.*
— **foreign** body, as if from, violent dry cough: Bell.[6]
— — — — — — cough: Tril.
— **grasp**, severe cough compels patient to grasp it; feels as if cough would tear larynx: *All. c.*[7]
— **irritation** to cough felt in: *Acon.*, *Alum*, Amm. c., Amm. m., Aph.,[1] Arn., Ars., Arum d.,[1] Bar., Bell.,[1] Bov., Bry., Calc., Canc. f.,[1] Canth., Carbo an., Carbo v., Caps., Caust., Cham., Chel.,[1] Cimic., Cina, Coc. c., Cocc., Coff., Colch., Coloc.,[1] Con., Dig.,[1] Dros., *Euphr.*,[1] For.,[1] Guare.,[1] Hepar, Hydr. ac., Iod., Ipec., Ign., Kali b.,[1] Kali c., Kali clc.,[1] Lach., Lachn., Laur., Mag. c., Mang., Manc.,[1] Merc. cor., Mez., Mur. ac., Naja,[1] Natr. c., Nux v., Olean., Osm.,[1] Puls., Rumex, Sabad., Sabin., Seneg., Sep., Sil., Spong., Staph., Stront., Sulph., Sul. iod.,[1] Tabac., Tarax.—5.
— **irritation,** above, cough from: Calad.[5]
— — in, afternoon cough: Ferr. iod., Phos.
— — — — 2 P. M., cough: Coca.
— — — back part, causes paroxysmal cough, evening in bed: Cocc.
— — — as from burning acid, cough: Aph.
— — — convulsive cough, at night in bed on waking: *Thuja.*

**LARYNX**, **irritation** in, dry cough: Bell., Kali iod., Sulph., Tabac.
— — — evening, on going out: Naja.
— — — extending to chest, cough from: Mez.[5]
— — — forenoon: Tongo.
— — — hacking cough: Osm., *Seneg.*
— — — short cough: Amm. c., Seneg.
— — — short asthmatic cough with painful sensation of contraction in chest: Amm. c.[7]
— — — from tickling, cough: *Brom.*, Cimic.
— — — and trachea, cough: Coca.
— — — upper part, during siesta, cough: Arnica.
— — to cough, from constriction and irritation: Naja.
— — — — after eating: Staph.
— — — — while eating: *Rumex.*
— — — — from pain: *Iod.*
— **itching** in, cough from: Ambr., Cact., Carbo v., Con.,[2] Dig.,[5] Laur., *Nux v.*, Puls.[5]—1.
— — — dry cough: Cact. gr.
— — — evening, cough: Carbo v.
— — — hacking cough: Laur.
— — — morning on waking, cough: Carbo v.
— — tickling in, convulsive cough: Calc. f
— — — — dry cough: *Bell.*
— — — — hacking cough: Calc. f.
— **lower part**, crawling in, cough from: Kreos.
— — — tickling in, cough from: Canc. f.
— — — — — morning after rising, causing hacking cough: Arn.
— **mucus** in, excites or agg. cough: Arg.,[1] Arg. n.,[1] *Brom.*,[5] Caust., Crot. t., Dulc., Euphr., Kali b., Kreos.,[1] Mang.,[1] Osm., Seneg.[1]—5.

**LARYNX**, **mucus**, balls of soap-like, accumulate, causing cough and expelled by it: Arg. n.
— — in, hacking cough: Cocc., Dig., Seneg.
— — membrane thickened or swollen, seems to cause short cough: Ery. a.
— — membrane feels as if it would be torn off with every cough: Osm.[1]
— — in, excites short cough: Seneg.
— — tenacious, causes hacking cough: Dig.
— **obstruction** of, after the cough: Arum. m.
— — — causing cough when eating fruit: Arg.
— **pain** in, causing cough: Acon., Ang., Bry., Calad., Euph., Grat.,[16] Kali c., Hepar, Spong.—12.
— — — when coughing: Arg., Bell., Bry., Carbo v., Chel., Osm.
— — — causes irritation to cough: *Iod.*
— — — one spot, agg. by pressure, cough, etc.: Hepar.[6]
— — — provoking coughing and hawking, agg. by pressure, on waking in morning, but toward noon giving place to a similar sensation behind upper third of sternum: Ferr.
— **painfulness** of, with danger of suffocating when touching or turning neck, or coughing or drawing a long breath: Bell.[6]
— **piercing pain** in, exciting cough: Acon.[2]
— **plug** (or valve) in, causes cough: Spong.[2]
— **pressure** upon, causes cough: *Chin.*, Cina, LACH., Rumex, Tarax.—5.
— — and roughness, a sensation between (which gradually becomes a tickling), causes cough: Tel.

**LARYNX**, **prickling** in, 1–4 A. M., cough: Bufo.
— — — 2 A. M., cough: Phos.
— **posterior part**, irritation to cough felt in, evening in bed: Cocc.
— — — mucus in, hacking cough: *Par.*
— uninterrupted provocation in, to a hacking cough; it does not disappear on coughing, but only when suppressing the cough: IGN.
— **rawness** in, causing cough: BROM., SULPH., Ziz.
— — — agg. by cough: Naja.
— — — dry cough: SULPH.
— — extending to sternum after cough: Osm.
— — when coughing: BROM., Kali c., Sep., Ziz.
— — and scraping in, dry cough: BROM.
— — — — — provoking dry cough in evening: BROM.
— — in upper part, when coughing, not when swallowing: ARGENT.
— **roughness** in, cough from: Bar., Carbo an., Dig., Kreos., *Mang.*,[1] Nux v.,[1] Sabad.—2.
— — and hoarseness, somewhat amel. by cough: Stann.[7]
— — and scraped feeling in, cough from: NUX V.
— — and pressure, a sensation between (which gradually passes into a tickling), causes cough: Tel.
— — dry cough brought on loud reading and talking, with painful dryness, roughness, and constriction of larynx: Mang.[10]
— **scraped** feeling and roughness in, cough from: NUX V.
— **scraping** in, cough from: Aur. m. n., *Bell.*, BROM., Bov., *Coc. c.*, Hepar, Hydr. ac., Led., Mang. ox., Nitr. ac., Nux v., Osm., Puls.,[5] Seneg.,[6] Til.—1.

**LARYNX**—(*Continued*).

— — in, cough evening: Bry.
— — — dry cough: *Bell.*, Hydr. ac., Led., Mang. ox.
— — — dry cough, night: Gamb.
— — — causes irritation to cough: *Con.*, Naja.
— — — night, dry cough: Gamb.
— — and rawness in, evening, dry cough: BROM., *Con.*
— — — — —, — cough: Chel.
— — and rawness in, provokes dry cough: BROM.
— — tickling in, dry cough: Mang. ox.
— — — — morning, dry cough: *Opium.*
— — — — — 3 A. M., dry cough: Opium.
— — — — bringing tears into eyes and causing dry cough: PULS.
— — and tickling in, dry cough, wakes him from sleep very early in morning with: Opium.
— **scratching** in, excites cough; Arg. n., Coloc., Dig.,[2] Kreos.,[2] Zing.—1.
— — — 1–2 A. M., dry cough: Zing.
— — — dry hacking cough: Zing.
— — — hard cough, amel. by discharge of mucus: Alum.
— — — shaking cough: Alumne.
— **shocks**, painful, in, from the cough: Hepar,[1] Sulph.[6]
— **side** of, stitches in right, on coughing: Aloe.
— — — tickling in left, causing cough: Tilia.
— — —, — — right, 7.15 P. M., cough: Ir. fœ.
— **smarting**, in causing cough: Zing.
— — — with cough: Ziz.
— **soreness** in, cough from: Upa.

**LARYNX, soreness** in, dry cough from: Zing.
— — — on coughing: ARG., Chin., Fl. ac., Sepia.
— — —, hoarse, racking cough, day and night, with profuse purulent expectoration: Ars. iod. (H. V. M.)
— — and rawness in upper part, when coughing, not when swallowing: ARGENT.
— — with or from cough: Amb., Brom., Carbo an., Carbo v., Chin., Ign., Kali c., Natr. m., Nux m.,[5] Phos.,[5] Rumex,[5] Sep., Stann.—2.
— **spasm** of, excites cough: Cupr.[2]
— — with cough: Bell., Laur.[2]
— **splitting** sensation on coughing: *All. c.*
— **stinging** in, or burning tickling, causing cough: Aph.
— **stitches** in, exciting cough: Aph., Bufo, Indigo, Kali c., Naja, Nit. d. s.
— — — dry cough in evening: Nit. d. s.
— — — — — night: Kali c.
— — — during cough: Kali c.
— — — worse evening, cough from: Bufo.
— — — paroxysmal cough: Indigo.
— — — on walking in open air, exciting cough: Ox. ac.
— **supports**, patient on coughing or swallowing involuntary supports the larynx: Dros.[7]
— **swelling** or thickening of the mucous membrane, sensation of, hacking cough: Ery. a.
— **swollen**, feeling cough from: Arn., Ox. ac.
— **tear**, hoarse cough seems to split and: *All. c.*[10]
— **tearing** sensation in, causes cough: Bov.

**LARYNX, tearing** pain on coughing, extreme pain on talking, even in a whisper, great hoarseness: Phos.[19]
— — See Larynx, torn.
— **thrusts** in, on coughing: Sulph.
— **tickling** in, excites or agg. cough: *Acon.*, Æsc. h., All. c.,[2] Alumn., Alum.,[2] Ambr.,[2] Anac., Ang., Ant. t.,[2] *Ars.*, Bar.,[2] Bell.,[2] Bor., Bov., *Brom.*, Bry., Calc.,[2] Carl., Caus., Cham.,[5] Chel., Cimic.,[5] Coc. c., Cad., Con.,[2] Cop., Crot. t.,[2] Dros.,[2] *Euphr.*, Eupion., Hepar, Indigo, Iod., Ipec., Iris, Kali b., Kali c., Lach.,[2] Lact.,[2] Laur.,[2] Led.,[5] Lob.,[2] Mag. c.,[2] Mag. m.,[2] Merc.,[5] Mez.,[5] *Natr. m.*,[2] Natr. s., Nitr. ac.,[5] Nitrum, Nux v.,[5] Olean.,[5] Osm., Ox. ac., Ph. ac., Psor.,[5] Rat., Rhus,[2] Rumex,[5] *Sabin.*, Sang., Seneg., *Sep.*, Sil., Spong.,[5] Staph.,[2] Sulph., Tarent., Tep., Til., Verb., Zinc., Zing.—1.
— — from abdomen to, excites cough: Sepia.[5]
— — in, which quickly changes to chest or stomach, etc.: Lach.
— — in, convulsive cough: Bad., Vin.
— — — — — night: Calc. f.
— — — day cough: Con.
— — — dry cough: Asaf., Am. m., Arg. n., *Brom.*, Carb. ac., Cimic., Coloc., Con., Cyc., Hydr. ac., Iris v., Kali b., Led., Mang. ox., Mez., Nit. d. s., Op., PULS., Ratan.
— — — eating apples temporarily relieves tickling in larynx, which excites hoarse, dry, or spasmodic cough: *All. c.*[2]
— — extending to middle of chest, cough from: *Ph. ac.*
— — in as from a feather exciting cough: Mez.
— — — — — — — causing an overpowering cough, in evening, before going to sleep: LYC.

**LARYNX, tickling** in, forcible cough: Lyc.

— — — cough after getting up: Sulph.

— — in, hacking cough: Acon., All. c., Ang., Carbo an., Coc. c., Dig., Ipec., Lach., Laur., Lob. s., Lyc., Rumex, Sabad., Spira., Spong.—10.

— — — causes inclination to cough: Cimic., Indigo.

— — insupportable, excites cough: Kali b.[2]

— — in, causes irritation to cough: *Brom.*, Cimic.

— **tickling-itching** in, dry cough: Bell.

— — — — excites short dry cough, evening after lying down, which cannot be suppressed: BELL.

— — — — convulsive cough, night: Calc. f.

— — — — hacking cough: Calc. f.

— **tickling** low in, dry cough, morning: Natr. s.

— — — — hacking cough, morning after rising: *Arn.*

— — in, extending to lung, cough: Sticta.[2]

— — — nervous cough: Lach.

— — — night, cough from: Sant., Sulph.

— — — — 7.30 P. M., irritation to cough: Cimic.

— — — — 11.30 P. M., cough waking, with expectoration of much tenacious mucus: Coc. c.

— — — — convulsive cough: Bad., Vin.

— — — — violent cough: *Cyc.*

— — — paroxysmal cough: Calc. f., Coc. c., Cyc., Lyc.

— — — racking cough, amel. by drinking water: *Opium.*

— **tickling-scraping** in, excites cough: Mang. ox.

— — — — dry cough, day: Con.

**LARYNX, tickling-scraping** in, dry cough 3 A. M.: Op.

— — — — dry cough, which wakes from sleep very early in morning: *Opium.*

— **tickling** in, excites severe cough: Chel.[2]

— — — shaking cough: Bry.,[1] *Olean.*[7]

— — — short cough: Agar., Graph., Iris v., Kali b., Laur., Led., Mez.

— — — short cough, when walking in open air: Ang.

— — — left side, cough: Phyto.

— — — right side, dry cough, evening: Ir. fœ.

— — — from smoking, dry cough: Coca.

— — — as from sugar dissolving, cough: Bad.[2]

— — — — — sulphur fumes, cough: *Lyc.*, Paris.[10]

— — itching in upper back part, evening after lying down in bed, dry cough: Bell.[7]

— violent tickling in, at 11.30 P. M., cough, waking, with expectoration of much tenacious mucus: COC. C.

— violent tickling and scraping in, bringing tears into eyes and causing dry cough: PULS.

— — tickling in, while walking in open air; larynx feels swollen, cough from: Ox. ac.[7]

— tickling and tingling in, amel. by coughing: Iod.[6]

— **tightness** of, dry cough in morning during menses with: Cap.

— **tingling** in, causes cough: Mag. m.,[1] Sep.[6]

— **titillating** in, when drawing in air, causing short cough, which shakes the whole body: Olean.[6]

**LARYNX torn** loose, sensation as if something were torn loose with cough; first day, then with profuse salty expectoration: Calc.[7]

— **torn**, sharp cough, feels as if larynx would be: Staph.[6]

— **touching** lightly agg. cough: China.[7]

— upper back part, tickling-itching in, evening after lying down in bed, dry cough: Bell.[7]

— upper part, irritation to cough: Coc. c.

— — — itching, cough, afternoon during siesta: Arn.

— — — rawness in, cough: Bell.

— — — rawness and soreness on coughing: Arg.

— — — roughness on inspiration or cough: Ziz.

— — — sensation as of sulphur fumes, cough: *Ars.*

— — — tickling, dry cough: Ipec.

— — — — causing desire to cough: Lob. s.

— — — — itching, cough, evening in bed: *Bell.*

**LAUGHING**, cough: *Arg.*,[1] Ars., Bry., *China*, Cupr., Dros., Kali c., Lach., Mur. ac., Nitr. ac., *Phos.*, Sin. n.,[1] *Stann.*, Zinc.—2.

—, aggravated, violent cough: Mur. ac.

**LEUCORRHŒA**, cough with: Nux v., Stann.

—, succeeded by hoarseness and cough: Con.[6]

**LIMBS**, jerking of, and want of breath, with dry spasmodic cough: Cina.[6]

**LIVER**, deep-seated, heavy pain in region of, worse from pressure, cough, laughing or deep inspiration: Psor.[22]

— painless twitching in, on cough: Lyc.

**LIQUIDS**, touching back part of mouth, cause cough: Amm. c.

— swallowing, night, convulsive cough: Sul. ac.

**LOINS**, pain in, with cough, vertigo, dyspnœa and shortings on chest: Kali. b.[10]

— — — and sides, with cough, compelling him to hold them: Kali. b.[6]

— stitches in, from, cough, before midnight: Rhus.[6]

**LOOSE** cough: Agar., Ant. t.,[5] Arum d., Asaf.,[5] Calc.,[5] Calc. acet., Carbo an., Carl., Cham.,[5] Chel.,[5] Chin.,[5] Chin. s., Cina,[5] *Coc. c.*, Cocc., Coloc. n., *Con.*, Cupr. n., Dig., Elaps, Eugen., Euph.,[5] Eupion, Feru., Graph., Hepar,[5] Hydras., Ign.,[5] Jabor., Kali c., Lapp., Linu., Mag. s., Merc., Merc. c., Natr. ars., Natr. c.,[5] Natr. s.,[5] *Phos.*, Podoph., Puls., Sabad.,[5] Secale, *Sepia*, Stil., Sulph., Tarent., Tel.—1.

— — breakfast, after: Coc. c.

— — chest, infiltration of lower: Kreos.

— — **drinking** cold water amel.: *Coc. c.*

— — from eating: Phos.

— — after eating; dry after drinking: Nux m.[7]

— — **evening** in: Mur. ac.

— — — 7 P. M: Spire.

— — — agg.: Eugen.

— — **loosen**, difficult to: Kreos., Osm.

— — **midnight**, about: Phos.

— — midnight, about, amel., sitting up: Phos.

— — **morning**: Coc. c., Fran., Hal., Mur. ac., Nux v., Stram., Sulp., Sulp. ac., Wies.—1.

— — **night**; Eug.

— — — agg.: *Sepia.*

**LOOSE cough, night**, first part, while up: Natr. ars.

— — sounds loose but no expectoration; agg. by exercise and entering a warm room: Brom.[7]

**LOUD** cough: Acon., Kali b.

— — from dryness of the throat: Stann.

— —, chronic, from stuffing sensation at the epigastrium, chiefly in the morning; has then a fit of coughing and expectoration of tough mucus, with lightness in head: Kali. b.[6]

— coughing during sleep: Lyc.[10]

**LUNGS, burning** in, when coughing: Arum tr.

— **irritation** of, causing cough: Lach.

— — to cough from pain in right, 8 P. M.: Dios.

— **loose** sensation, as of something loose and fluttering in, during cough: Phos.

— **pain** in, from cough: Asc. t., Tarent. (1), Zing.

— — — lower part of, from cough: Tarent.

— — — in right, causing irritation to cough, 8 P. M.: Dios.

— — — with dry, hacking cough and difficult breathing: Zing.[10]

— **rawness** in left from cough, morning: Chin. s.

— **rested** against back, sensation as if, on coughing: Sulph.

— **ruffling**, wandering, contused, after coughing: Gad.

— **soreness** in left agg. by coughing, deep inspiration, or sneezing: Chel.

— — evening, on coughing: Ustilago.

— — morning, on coughing, after deep inspiration and yawning: Gal. ac. (r).

**LUNGS, spasm** of, with blue face and cough: Opium.[7]

— **stitches** in, from cough: Clem.

— — — lower part, agg. by cough: Thuja.

— **strained** sensation in, after coughing: Calc. s.

— **tearing**, as if torn out, when coughing: Elaps.

— **tickling**, as from a sensation of something loose and fluttering in, during cough: Phos.

— **upper part** of, pain in (worse on right side), when coughing: Elaps.

— — — torn out sensation when coughing: Elaps.

— **weight**, cough as from a: Angust.

**LYING excites** or agg. cough: All. c.,[2] Ambr., Amm. m., Apis, *Ars.*, Bell., *Bry.*, Calc., Caps., Carbo v., Cham., Cinnab.,[1] Cocc., Coc. c.,[2] Con., Dros., Euphr., Ferr., Hyos., Ign., Ipec., Lach., Lact., Lith. c., Lyc., Mar., Meph., Merc., *Mez.*, Natr. m., Nicc., Nitr. ac.,[11] Nux v., Par.,[1] Petrol.,[11] Phos., Ph. ac., Puls., Rhus, Rumex,[2] Ruta, Sabad., Sang., Sep., Sil., Staph., Stann., *Sulph.*,[11] Thuja, Verb.—5.

— **afternoon**, 2 P. M., hacking cough: Laur.

— — 3 P. M., hacking cough: Calc. ph.

— **amel**. cough: Acon., Amm. m., Euphr., Mang., Sep., Zinc.

— on **back**, agg. cough: Amm. m., Iod.,[10] Kali b.,[11] *Natr. m.*,[11] *Nux v.*, *Phos.*, Plumb.,[7] Rhod.,[10] Rhus,[10] Sil.—5.

— on **back** or right side, agg. irritable cough: Phos.

— — — agg. dry cough: *Phos.*

— — — dry cough, midnight: *Nux v.*

**LYING** on **back**, dry cough going off when turning on side: Nux v.[6]
— — — causes irritation to cough: Natr. m.
— — — relieves cough: *Mang.*
— — — partially relieves dry cough, with nervous excitability: Acon.[7]
— in **bed**, agg. or excites cough: Anac., Ant. t., Aralia,[10] Ars., Bry.,[6] Cact., Cham., Coc. c.,[2] Crot. t., Dolich., Dros., Euphr., Ferr., Hepar, Ign., Indigo, Iodof., Kreos., Lach.,[10] Lachn., Mag. c., Mag. m., Meph.,[2] Natr. m., Nux v., PHOS., Puls., *Rhus*, Samb., Sang.,[2] Sep., Squil., Staph., Verb., Vit.—5.
— — **bed**, cough when lying in bed, must sit up, or sleep in chair from sense of suffocation: Crot. t.
— **constant** cough while, amel. on sitting up: HYOS., PULS., Rhus, Sang.
— **day**, during, amel. cough: Sepia.
— **down**, cough almost only when first lying down, day or evening; was obliged to sit up and cough it out, when had rest: CONIUM.
— — after, cough prevents sleep and wakens him about midnight, with same clicking down back, which irresistibly irritates to violent shocks in head; ceases after loosening a small lump of mucus, which is swallowed: APIS.
— — cough commences morning on rising, and lasting until lying down: *Euphr.*[7]
— — constant cough in evening after, disappears on sitting up, but returns on lying down: HYOS., PULS.
— — deep cough ceases on, and better at noon: MANG.[1]

**LYING down**, evening cough directly after, has to sit up: Ars.[7]
— — — cough most severe in, after: SEPIA.
— — — short hacking cough in, after: SEPIA.
— **dry** cough from: Cinnab., *Kali br.*, Phos.
— after **eating**, agg. short cough: Tereb.
— in **evening**, cough: Ars., Dros., Ferr., Graph., Lach., Mar., Natr. m., Staph.
— — — amel. dry cough: Amm. c., Zinc.
— — — constant cough: PULS.
— — — dry cough: *Nux v.*
— — — hacking cough on: SANG.
— — — agg. hacking cough: Sil.
— — — amel. hacking cough: Amm. m.
— — — hollow cough: Lact.
— — — paroxysmal cough: *Nux v.*
— — — paroxysmal cough, 11 P. M.: *Rumex.*
— — — scraping cough: Bry.
— — — violent cough: *Kali c.*
— **evening** while, a violent cough, must rise up: Lith. c.
— **hacking** cough: Par., Vesp.
— — — evening: SANG.
— — — agg. evening: Sil.
— — — amel. evening: Amm. m.
— with **head low**, agg. cough: Amm. m., CHIN., Hyos., Puls., Samb., Spong.—5.
— with head raised amel. cough: CHIN.[5]
— **irritation** to cough, evening: *Ign.*, *Mez.*, Sulph.
— **long** in one position (or sitting) agg. cough: Con., Ph. ac.—2.
— **midnight** dry exhausting cough, lying on back, worse after: Nux v.
— — after sleeping and just before, cough: *Aralia r.*

**LYING night** agg. dry cough: Kali br.

— **provocation** to hacking cough in larynx, evening: *Ign.*

— **severe** cough, especially after, and on sleeping: Apis.[7]

— on **side**, agg. or excites cough: Bar., Carbo an., Kali c., Kreos., Lyc., Merc., Phos., Puls., Seneg., Sep., Stann., Sulph.—2.

— on side, midnight, amel. dry, exhausting cough; agg. lying on back: *Nux v.*[1]

— left side, agg. or excites cough: Acon., Bar., Bry., Ipec., Lyc., Merc., Par.,[1] Phos., Puls., Rhus, Rumex, Seneg., Sep.—5.

— painful side, agg. cough: Acon.[2]

— on right side, agg. or excites cough: Acon., Am. m., *Carbo an.*, *Cina*, Ipec., *Stann.*—5.

— on right side, night, dry cough: *Carbo an.*

— on right side or back, agg. irritable cough: Phos.

**MAMMÆ**, cough with pain under left mamma through to left scapula: Comocladia.[14]

— short dry cough excited by a tickling sensation behind upper half of sternum, with dull aching in left mammary region when sitting inclined forward, at 11 A. M.: *Rhus.*

**MANUAL labor**, cough from: Led., Natr. m.[2]

— See Exertion.

**MATCH**, burning sensation as from fumes of a, in throat, irritation to cough from: Aml. n.

**MEDITATION**, cough from: Arn., Cist., Ign., *Nux v.*—12.

**MEALS**, swelling of stomach and cough after: Kali b.[6]

— violent spasmodic cough, commencing with gasping for breath and continuing with repeated crowing inspirations, till the face becomes black or purple and the patient exhausted; agg. at night and after a meal: *Coral.*[2]

— See Eating, Drinking, etc.

**MEASLES**, cough after: Ant. cr., Bry., Camph.,[7] Cham.,[6] Coff.,[7] Con.,[12] Dros., Dulc.,[7] Eupat. per., Gels.,[7] Hyos., Ign., Nux v.[12]—**2.**

— **after**, catarrhal cough: Cham.[6]

— — chronic mucous cough: Dulc.[7]

— — croupy cough: Gels.[7]

— — hoarseness and violent dry cough: Camph.[7]

— **during**, dry cough: Coffea.[7]

**MEMBRANE**, tormenting cough gets nothing up; sometimes feels as if a tough membrane were moved about but would not loosen: Kali c.[7]

— whizzing cough, with sensation as if the mucous membrane were too dry: Lauro.[10]

**MENSES, at beginning of**, exhausting cough: Phos.

— — — — hacking cough: *Phos.*

— **before** cough, evening, in bed, amel. by sitting up: *Sulph.*

— — and during, cough fatiguing chest early in morning and by day (not at night): Graph.[6]

— — — — evening, cough: Zinc.

— — — — morning, cough: ZINC.

— **during**, agg. of cough: Amm. m., Graph., Rhod., Zinc.—5.

— — bursting pain, as if coryza were coming on during menses, on coughing, sneezing, or stooping: Natr. m.[20]

— — coughs up mucus as thick as lard: Thuja.

— — dry cough in morning and tightness of larynx: Copaiva.

— — dry cough, with profuse sweat: Graph.

**MENSES**, during, hoarseness and dry morning cough: *Copaiva.*

**MENTAL emotion** agg. cough: Acon., Ant. t., Asar., Bry., *Cham.*, *Cist.*, Lach., Nux v., Op., Rhus, Staph.—5.

— **exertion** agg. cough: Arn., Asar., Cist., Colch.,[5] Ign., Nux v.—2.

— — agg. whooping cough: Arn., Ign., Nux v.—8.

— — reading, agg. whooping cough: Cina.[8]

**METALLIC** cough: Eupion.

**MILK** agg. cough: Ambr., Ant. c., Ant. t., Brom., Kali c., Sul. ac., Zinc.

**MIDNIGHT**, cough about: Amm. c., Ant. t., Ars., Bar., Bell.,[11] Bry., Calc. a.,[1] Caust., Cham., Coff., Dig., Hepar, Kali c., Manc.,[1] Mag. c.,[5] Mag. m.,[5] Mez., Nitr. ac., Nitr., Nux v., Phos., Rhus r., Samb., Zing.[1]—16.

— **about**, cough **amel.**: Hipp.[5]

— — **bed**, cough on going to: *Sepia.*

— — **choking** cough about: Ruta.

— — **dry** cough: Grat.

— — — — till daybreak: Nux v.

— — — — agg. lying on back, amel. lying on side: *Nux v.*

— — **exhausting** cough, lying on back: Nux v.

— — **irritation** to cough about: Coffea.

— — **loose** cough about: Phos.

— — **paroxysmal** cough about: *Cham.*, Naja.

— — — — — something seems to rise in throat as if it would choke him: *Cham.*

— — **persistent** cough, lying on back, amel. lying on side: *Nux v.*

— — on **waking**, cough, amel. by expectoration: Sulph.

— cough **after**: *Acon.*, Amm. c., Ant. t., *Ars.*, Bar.,[11] *Bell.*, Bry., *Calc.*, Caust., Cham.,[11] Chin., Cocc., Coff.,[11] Digit, Dros., Grat.,[11] Hepar, Hyos., Iod., Kali c., Lyc., Mag. c., Mang., Merc., Mez., Nitr. ac., *Nux v.*, Phos., Ph. ac., Rhus, Rumex, Samb., Spong.—5.

**MIDNIGHT**—(*Continued*).

— **after**, compare with Morning.

— — cough **agg.**: Mezer.

— — **dry** cough: *Ars.*, *Calc.*

— — **short** cough: Acon., *Ars.*

— cough **before**: Alum.,[5] Ant. t., Arn., Ars.,[5] Bar., Calc.,[5] Carbo v., Caust., Ferr., Graph.,[5] Hepar, Kali c.,[5] Led., Lyc., Mag. c., Mag. m., Mezer., Mosch., Mur. ac., Natr. m.,[5] Nitr. ac., Nux v., Phos.,[5] Puls., Rhus, Rumex, Sabad., Sep., Spong., Squil., Stann., Staph., Sulph., Sul. ac., Verat., Zinc.—2.

— **before**, compare with evening cough.

— — cough at 11 P. M.: Ant. t., Rumex, Verat.—5.

— — cough **agg.**: Osm.

— — **anxious** cough, waking: Rhus.

— — **dry** cough: Nitr. ac., Rhus.

— — — — especially in evening in bed, till midnight, frequently with nausea and bitter vomiting: Sepia.[10]

— — — — in sleep: Nitr. ac.

— — cough worse from evening till: Phos.

— — irritation to cough, scanty expectoration: STANN.

— — paroxysmal cough, causing pain in head, from crawling in wind-pipe near supra sternal fossa: Apis.

— — paroxysmal cough, amel. by loosening and swallowing mucus: *Apis.*

— — short cough, waking: *Rhus.*

— — stitches in loins with cough Rhus.[6]

**MOANING** with the cough: *Bell.*, Cina, Podo.—5.

**MOIST** cough: Acet. ac., Am. cau., Nux m., Piloc., Tarent.

**MOON**, **full**, cough agg. at: Nitrum,[1] Sabad.[5]

— **new**, cough agg. at: Sabad., Sil.—5.

**MORNING** cough: Acon.,[1] Alum., Amm. c., Amm. m., Anac.,[1] Ang., Ant. t.,[1] Arn., Arg., ARS., Arum d.,[1] Aur., Bar., Bell., Bov., Bry., Calad., Calc., Canc. f.,[1] Canth., Carbo an., Carbo v., Caust., Cham., Chel., CHINA, Cina, Coca,[1] *Coc. c.*, Cod.,[1] Colocn.,[1] Coral., Crot. t., Cupr., Dig., Dros., Erio., EUPHR.,[1] Ferr., Grat., Gymno., Hepar, Ign., *Iod.*, Ipec., KALI B., Kali c., Kreos., Lach.,[11] *Led.*, *Lyc.*, Mag. c., *Mag. s.*, Mang., *Meph.*, Merc., Mur. ac., Naja, Natr. c., *Natr. m.*, Natr. s., Nitrum, Nux m., NUX V., Osm.,[1] Par., Phos., Ph. ac., PULS., Pyrus,[1] Rhod., Rhus, Sars., *Selen.*, Seneg., Sep., Sil., Spig., Squil., Stann., Staph., *Sulph.*, Sul. ac., Sum., Tabac., Thuja, Verat.—5.

— cough at 1 A. M.: Coc. c.

— — — 1–2 A. M., from scratching in larynx: Zing.

— — — 1–4 A. M., pricking in larynx: Bufo.

— — — 2 A. M.: Cocc.,[5] *Opium*,[1] Phos.,[1] Rumex.[5]

— — — 2 A. M., amel. by drinking water: Glon.

— — — 2–3.30 A. M., paroxysmal cough: Coc. c.

— — — 2 or 3 A. M.: Ant. t., Merc.

— — — 3 A. M.: Amm. c.,[5] Bapt.,[6] Cupr.,[5] KALI C.,[5] Mag. c.,[1] Mur. ac.,[10] Nitrum.[5]

— — — 3 A. M., tickling and scraping in larynx: Opium.

**MORNING** cough at 3 A. M., cough wakens at night with stupefying headache: Nitrum.[7]

— — — 3–4 A. M.: *Amm. c.*,[10] Cainca, *Kali c.*,[7] Lyc.[1]

— — — 4 A. M.: Anac.,[10] Nitr. ac.,[10] Nux v.[5]

— — — 4 A. M., waking one: Nitr. ac.[10]

— — — 5 A. M.: Arum t.,[1] Kali c., Rumex.—5.

— — — 6 A. M., severe cough about: *Alum.*

— — — 6.30 A. M., from tickling low in trachea, a deep cough: Dios.

— — — 6–7 A. M., Arum t., Calc. ph.

— — — 8 A. M., Ham., Ol. an.[10]

— — — 8–9 A. M.: Sil.

— — — 9 A. M.: Sep., Tarent.

— — — 9–12 A. M.: Staph.

— — — 10 A. M.: Aqu. petr.

— — — 10 A. M., from rawness in air passages while lying: Coc. c.

— — — 10–12 A. M.: Natr. m.[5]

— — — 11 A. M., dry cough from tickling behind upper half of sternum, when sitting bent forward: *Rhus.*

— — — 11 A. M., from rush of blood to chest: Raph.

— cough **agg.** in: *Alum.*, Bov., Chin. s., Dir., Sil., Tellur., Zing.

— — — on waking: *Sil.*

— — after **bath**: Calc. s.

— — in **bed**: Amm. c., Bry., Nitr., Rhus.

— — before **breakfast**: Murex.

— — from mucus in upper **chest**: Plumb.

— — tickling in chest, on entering warm room from cold air: Bov.

— — **cold**, on rising: Carbo v.

— — till evening: Sil.

— — from deep inspiration, on lying down: Ipec.

**MORNING** cough, itching in **larynx**, on waking: *Carbo v.*
— — pain in larynx, on waking: Ferr.
— — dryness in **pharynx**: Phyto.
— — **throat**, dryness in: Carbo an.
— — — irritation in left side of: Dios.
— — — painful spot in: Chin. s.
— — — tickling in, after rising: Alumn.
— — — rawness in: Ol. an., Phos.
— — **trachea**, irritation in bed: Sulph.
— — — tickling in: Bov., Sep.
— — — — — on rising: Chin. s., FERR., Hal., Indigo, Sil.
— — — — — on waking: Ail., Cod., Sulph., Tarent.
— **choking** cough on rising: CINA.
— — — agg. morning and evening: Stram.
— **chronic** cough: Iod., Lyc.—12.
— **constant** cough in: Cupr. s.
— **convulsive** cough in: Natr. ars., Thuja.
— — — in bed: Ferr.
— — — on waking: Thuja.
— **deep** cough in; Dios.
— — — from the trachea, with expectoration of yellow mucus: Ang.
— — — from tickling low in throat, 6.30 A. M.: Dios.
— **dry** cough in: Agar., *Alum.*, Amm. m., Ant. t.,[11] Bar., Bov., Brom., Bry., China,[11] Coc. c., Con.,Cop., Dios., Grat.,[11] Gymn.,[12] Lyc., Mag. s.,[12] Mim., Mit., Natr. c., Natr. s.,[12] Nux v., Puls.,[10] Rhod., Sang., Squil.,[12] Stann.,[11] Sulph., Sul. ac., Tabac., Tarent., Thuja, Verat.—1.
— **dry** cough agg. in: Bor., Brom., *Mosch.*, Stann.

**MORNING, dry** cough agg. early in: *Alum.*, Amm. m., *Ant. cr.*, China, Grat., Lyc., Nux v., Rhod., *Stann.*, Sul. ac., Verat.—11.
— **dry** cough in, and tightness of larynx, during menses: Copaiva.
— — — — — hoarseness, during menses: *Copaiva.*
— — fatiguing dry cough in, after waking, obliging one to sit up, which relieves: Mag. s.[6]
— — severe dry cough, mostly in, with retching and desire to vomit, and sensation as if stomach were turned inside out: *Puls.*[10]
— — severe dry cough about 6 A. M.: *Alum.*
— **dyspnœa**, especially in morning with cough, and expectoration of white mucus as tough as pitch: Kali b.[6]
— **exhausting** cough: Rhodo., Sulph.
— — — agg. in, and evening on going to sleep: Lyc.
— — — after waking: Mag. s.
— — — on waking: Thuja.
— **gagging** cough in, after rising: *Cina.*[7]
— **hacking** cough in: All. c., Calc., Cina, Iris v., Kali c., Kali iod., Mit., Nitr. ac., Ol. an., Sil., Sum.—1.
— **hacking** cough agg. in: Sum.
— — — — on waking: Sil.
— — — from mucus: Laur.
— — — after rising: Arg., Par.
— — — on speaking: Sum.
— — — dryness in throat: Ant. t., Mang.
— — — itching in throat: Ant. t., *Con.*
— — — tickling in throat: Sum.
— — — — low in trachea, after rising: *Arn.*

**MORNING**, **hard** cough in, eyes fill with tears, mucus so hard to detach: Cina.[6]
— **hoarse** cough, worse morning and evening: Caust.
— **hollow** cough, in bed: Phos.
— — — especially at night and in the morning, with tightly adherent mucus in chest, in which there is a sticking pain, very sore and ulcerated, with and without coughing, with stopped coryza and stoppage of the nose: *Caust.*
— **hollow** cough, after rising: Cina.
— — — on waking: IGNAT.
— **irritable** cough, after rising: Arg.
— **irritation** to cough in: Dios., Meny., Ol. an., Thuja.
— — — — agg., in bed: *Caust.*
— — — — on deep inspiration: *Cori. rus.*
— — — — after rising: Alumen.
— **loose** cough in: Coc. c., Fran., Hal., Mur. ac., Nux m., Stram., Sulph., Sul. ac., Wies.
— **loud** cough, chronic, from stuffing at the epigastrium, chiefly in the morning; has then a fit of coughing and expectoration of tough mucus, with lightness in head: Kali b.[6]
— **paroxysmal** cough in: Agar., *Ant. cr.*, Coc. c., Nux v., Ol. jec., Sang., Sul. ac.
— — — agg. in, eating warm food: amel. by rinsing mouth with cold water and by cold drinks: Coc. c.
— **racking** cough, on waking: Caust.
— — — much cough, especially in the morning, cough racking as in consumption, with much expectoration coming deep from lungs; the cough could not be prevented by changing the position, after waking, since the rattling in the chest increased; expectoration in large quantities, difficult to loosen; during the first days the paroxysms of cough, with pain behind the sternum occurred especially at night: *Chelid.*

**MORNING**—(*Continued*).

— **on rising**, cough: *Ars.*, Chin. s., FERR., Hal., Indigo, Sil.
— — — cough, and continues until lying down again can; scarcely get breath; none at night, worse from smoking, better from eating: *Euphr.*[7]
— — — dry cough: *Carbo an.*, Grat.
— — — See Rising from Bed.
— **after rising**, cough: Euph., Lach., Nitr. ac., Staph., Thuja.
— — — cough, from tickling in throat: Alumen.
— — — cough with expectoration of transparent mucus, and torn sensation in middle of sternum: *Phos.*
— — — choking cough: CINA.
— — — dry cough: Alum., Ang., Bar., Bov., Carbo an., Dig., Natr. slfc.
— — — gagging cough: Cina.[11]
— — — hacking cough: Arg., *Arn.*, Par.
— — — — — tickling low in trachea: *Arn.*
— — — hollow cough: Cina.
— — — irritation to cough: Alumen.
— — — short cough: Arn.
— — — tickling cough: Alumen.
— before **rising**, soreness in chest, with violent cough, and expectoration of clotted blood: NUX V.
— **violent**, sudden cough in, with stitches in side at every cough, with expectoration: SQUIL.

**MORNING**, after **waking**, exhausting cough: Mag. s.

— — — paroxysmal cough: RUMEX.

— — — tickling cough: Carbo v.

— on **waking**, cough: Ail., Cod., Sulph., Tarent.

— — — — agg.: *Sil.*

— — — convulsive cough: Thuja.

— — — dry cough: Caust., Ign., Mag. s., Sil.

— — — exhausting cough: Thuja.

— — — hacking, agg. on: Sil.

— — — hollow cough: IGN.

— — — racking cough: Caust.

**MORTIFICATION**, cough after, with stitches in side and frothy, bloody expectoration: Puls.[8]

**MOTION**. See Movement.

**MOUTH**, bleeding from, with cough: Dros., Ipec., Merc., Nux v.—5.

— **dry**, with cough: Cocc., Laur.[5]

— **herpetic** eruptions around, with cough: Ars., Natr. m., Rhus.[17]

— **liquids**, touching back part of, excite cough: Amm. c.

— **mucus** in, causes hacking cough: Sul. ac.

— **sensation** as of a finger thrust into back part of, excites cough: Merc. c.

— **pain** in, with cough: Mag. s.[16]

— **tickling** in roof of, dry cough: Nux v.

— See Rinsing.

— **roughness** and scratching on roof of, and in trachea, hollow, deep, spasmodic cough: Digit[7]

— disagreeable **taste** in, with cough: Caps.[16]

— sour taste in, when coughing: Cocc.[6]

— **water** in, with cough: Ambr., Ars., Bry., Lach.[16]—6.

**MOUTH**, **water in**, continued dry cough early in morning, with discharge of water, like waterbrash, from mouth: Bry.[6]

— — deep dry cough with flow of water in mouth, and subsequent scraping in throat: Ambr.[6]

**MOVEMENT**, cough agg. after: Ars., Zinc.—5.

— **excites** or agg. cough: Arn., *Ars.*, Bar., Bell., Brom., Bry., Bufo[1], Calc., Carbo v., *China*, Dros.,[11] Eupat. per.,[2] Ferr.,[1] Iod., Ipec., Kali c., Kreos., Lach.,[11] Laur., Led., Lyc.,[12] Merc., Mez., Mosch., Mur. ac., Natr. m., Nitrum, *Nux v.*, *Phos.*, Sep., *Sil.*, Spong., *Stann.*, Squil., Staph., Sul. ac.—5.

— **amel.**, violent dry cough, felt while lying or sitting: Phos.[6]

— **commencement** of, cough: Nitr. ac., Plan.,[1] Sil.—5.

— of **arms**, excites cough: Ars., Calc., Ferr., Kali c., Led., Lyc. (r.), Natr. m., Nux v.—12.

— of **chest**, excites cough: Anac., Bar., Cocc., CHINA, Dros., *Lach.*, Mang., Merc., Mur. ac., Natr. m., NUX V., *Phos.*, Sil., STANN.—11.

— in open air, agg. hacking cough: Osm.

— on, 1 P. M., cough: Natr. s.

— on, 4 P. M., cough: Calc. f., Kali b.

— **short** cough, from: Carb. ox.

— **violent**, excites cough: Stann.[5]

**MUCOUS** cough: Kali c.

— — morning and night, after rising: Sulph.

**MUCUS**, discharge of, amel. hard shaking cough: Alumen.

— **balls** of soaplike, accumulate in larynx, causing slight cough: Arg. n.[6]

— **expectoration** of tenacious amel. forcible cough: *Phos.*

**MUCUS**, **hacking** cough from, morning: Laur.
— **swallowing**, amel. paroxysmal cough, before midnight: Apis.
**MUSIC** excites coughing: Ambr., *Calc.*, Cham., Kali c., Kreos., Ph. ac.—2.
**NARES**, sensation as of an acrid fluid through posterior, excites cough: Kali b.[12]
**NAUSEA**, with cough: Ant. t., Ars., Aspar.,[1] Bry., Cajup.,[1] Calc.,[11] Caps.,[1] Coloc.,[1] Dros., Elaps,[1] Hepar, Hydras.,[1] Ignat., Ipec., Iod., Kali b., Kali c.,[11] Lach.,[11] Merc., *Merc. sol.*,[1] Natr. c., Nitr. ac.,[1] Petro.,[11] Ph. ac., Pic. ac.,[1] Puls., Ruta, Sabin., Sars., Sep., Squil., Thuja,[1] Verat.[11]—5.
— **agg.** by dry cough: Nux v.
— excites cough: Bry., Caps.
— and retching with cough: Verat.
— in pit of stomach, excites cough: *Ign.*
**NECK**, **extending** or stretching neck excites cough: *Lyc.*
— pain in **glands** of, on coughing: Natr. m.
— pain in **muscles** of, on coughing: Bell., Lact.
— pain in **nape** of, with cough: Alum., Bell.—12.
— violent, short dry cough with sneezing and tearing, lancinating, pinching pain from nape of neck to right axilla: Alum.[6]
— violent pressure in nape of neck during a fit of coughing, as though it would break: Bell.[6]
— drawing pain in side of chest extending up to neck, on coughing: Caps.[6]
— **pricking** in, with dry cough: Vich.
— on **touching** the, under right ear, in afternoon, a cough: Fago.
**NECK**, on **touching**, painfulness of larynx with danger of suffocation when touching or turning neck, or coughing or drawing a long breath: Bell.[6]
— Compare with Larynx, Throat, etc.
**NERVOUS** cough: Plumb.
— — from tickling in larynx: Lach.
— — when any one enters the room: Phos.[7]
**NIGHT** cough: Acon., Agar., Alum.,[2] *Ambr.*, *Amm. c.*, *Amm. m.*, Anac., Ant. t.,[2] Apis, Aqu. petr., Arg. n., Arn., Ars., Asar.,[16] Aur., Aur. mur., *Bar.*, Bell., Bism.,[5] Bor., Bry., Calad., Calc., *Caps.*, Carbo an., Carbo v., Cast.,[16] *Caust.*, Cham., *China*, Cina, Cocc., Coc. c.,[5] Coff., *Colch.*, Coloc.,[5] Con., Coral.,[2] Cupr., *Cycl.*, Dig.,[12] Dros., Dulc.,[2] Eugen., Ferr., Gamb., Graph., *Grat.*, Guai.,[5] Hepar, Hipp.,[5] Hyos., *Ign.*, Indigo, *Ipec.*, Kali b.,[16] Kali c., *Kreos.*, Lach., Lact.,[2] Led., Lepi., Linu., *Lyc.*, *Mag. c.*, *Mag. m.*, Meph.,[5] Merc., *Mez.*, *Natr. m.*, Natr. s., Nicc.,[5] Nitr. ac., Nitrum, *Nux v.*, Ol. an.,[12] Ol. jec., Op., Par., Petro., Phell.,[12] Phos., Puls., Rhod., *Rhus*, Rumex, Ruta, Sabad., Sang.,[2] Seneg.,[12] Sep., Sil., Sol. t. æ., Spig.,[5] Squil.,[5] Stann.,[5] Staph.,[5] Sticta,[2] Stront.,[5] Sulph.,[1] Thuja, *Verat.*, *Verb.*, Vich., Zinc. —1 and 11.
— Compare with Midnight, before and after.
— cough **agg.** at: Anac., Ars., *Caps.*,[12] *Cham.*,[12] *Chel.*, Gam., Op., *Natr. s.*,[12] *Nitr. ac.*, Puls., Stront., Tarent.—1.
— cough agg. at nightfall: Erio.
— — — — 5–9 P. M.: Caps.

**NIGHT** cough agg. at 8–11 P. M.: Natr. m.

— — — violent convulsive cough (whooping cough) caused by tickling in pharynx; agg. at night and after eating; after the attacks yawning and sleepiness: *Anac.*[10]

— — — much cough, especially in the morning; cough racking, as in consumption, with much expectoration coming deep from lungs; the cough could not be prevented by changing position, after waking, since the rattling in the chest increased; expectoration in large quantities, difficult to loosen; during the first days the paroxysms of cough, with pain behind sternum, occurred especially at night: *Chelid.*

— — — hollow cough, especially at night and in the morning, with tightly adherent mucus in chest, in which there is a sticking pain, very sore and ulcerated, with and without coughing, with stopped coryza and stoppage of nose.—*Caust.*

— **cough**, till 4 A. M.: Sil.

— — commencing at 10 P. M., and returning every few minutes: Bell.[6]

— — **air passages**, from irritation in: Cham.

— — — from tickling in: Sant.

— — in **bed**: Amm. m., Arg. n., Psor.

— — — on falling asleep: Petro.

— **bed**, cough at night in bed, compelling one to spring up and assume an erect posture at once: BRY.

— — when going to bed, coughing with heat in head and face, and cold hands: Sulph.[6]

**NIGHT cough, breath**, from want of: Aur., Coloc.

— — — irritation: Com.

— — **chest**, with sore pain in, where expectoration is detached: Lyc.[6]

— — — with pricking in: Zinc.[6]

— — **larynx**, from tickling in: Sant., Sulph.

— — — — scraping in: *Cycl.*

— —, coughs only at night: Lach.,[10] Merc.,[5] Phos.,[10] Sulph.[13]

— — **sit up**, cough in evening or at night, with suffocation, obliging him to sit up: *Ars.*[6]

— — — cough at, obliging him to sit up and hold his head with both hands: Nicc.[6]

— — in **sleep**: Calc.

— — **stomach**, from irritation in: *Merc. s.*

— — **throat**, from constriction in, during sleep: Nitr. ac.

— — — from irritation in: *Ambra.*

— — — — rawness in: *Anac.*

— — — — roughness and scraping in, lying on right side: Sil.

— — — — scraping in: Con.

— **convulsive** cough: Agar., Mag. c., Sulph.

— — — in bed: Tarent.

— — — from irritation in larynx, on waking: *Thuja.*

— **dry** cough: *Acon.*, Agar., Aloe, *Alum.*, *Amm. c.*, *Amm. m.*, Arg. n., Ars.,[11] Aur. mur., *Bell.*, Bry., *Calc.*, Caps., *Carbo v.*, Cent., *Cham.*, China, Coc. c., Gamb., *Graph.*, Grat., HYOS., *Kali c.*, Linu., Lyc., *Mag. c.*, *Mag. m.*, Mag. s., Mang. ox., *Merc.*, *Mez.*, Natr. m., *Nux v.*, Ol. an., Op., *Petro.*, PHOS., PULS., Rhod., *Rhus*, *Sabad.*, Sil., Sol. t. æ., Spira., SPONG., Squil., Stront., SULPH., Tarent., *Verat.*, *Verb.*, Vich.—1 and 12.

**NIGHT**, **dry** cough, **agg.** at: AMM. c., Cham., Coloc., *Kali br.*, Op., Stront., Tabac.—1 and 12.

— — — **amel.**, by day or by night, relieved by detaching and raising a little mucus: *Guai.*

— — — — at night: Bar.

— — — — on sitting up: HYOS., PULS., *Sang.*

— — — — by smoking: Tarent.

— — — **air-passages**, from tickling in: Coc. c.

— — — in **bed**: Arg. n., Sulph.

— — — **chest**, from intense pain in: Kali c.[6]

— — — — from tickling in upper anterior: Polyg.

— — — titillating dry cough, with sort of spasm in chest, at night: Sepia.[6]

— — — and **day**: Bell., Euph., Ign., Lyc., Spong.—11.

— — — and in **evening**, with sensation as if small bubbles were breaking in the chest: Sulph.[6]

— — — **hoarseness** at night, with dry cough and blood-streaked saliva: Arg. n.[6]

— — — **hypochondrium**, stitches in left, with: Amm. m.[6]

— — — **larynx**, from scraping in: Gamb.

— — — — from sticking in: Kali c.

— — — **lying** on right side: *Carbo an.*

— — — **menses**, during: Zinc.

— — —, **severe**, with tearing pains in head and stitches in chest, followed by palpitation of heart for a few minutes: Cupr. acet.[6]

— — — **throat**, with burning in: Castor.[6]

— — — — from tickling in: Crot. c.

**NIGHT**, **dry** cough, with **vomiting** and sweat, as from anguish; has to get up: Sil.[8]

— — — **waking** one: Kali c., SULPH.

— **exhausting** cough: Natr. c., Rhod.

— — — in bed: Tarent.

— **hacking** cough: Graph., Kali c., Mag. s., Natr. m.

— — dry cough from tickling in larynx, or dryness in pharynx, worse at night as soon as he lies down: Phyto.[7]

— — — from tickling in pharynx: *Sil.*

— **hard** cough: Apoc. c.

— **hoarse** cough: Verat.

— — racking cough, day and night, with profuse purulent expectoration; soreness in larynx: Ars. iod.

— **hollow** cough: *Phos.*, Spong.

— — — agg. morning and night: *Caust.*

— **incessant** cough at, cannot sleep: SEPIA.

— **irritable** cough agg. at: *Codeinum.*

— **irritation** to cough: Ferr.

— — — — on waking: Thuja.

— **laid**, constant cough whenever the child is laid down, particularly at night: *Sepia.*

— **loose** cough: Eugen.

— — — agg. at: Sepia.

— — — first part of night, while up: Natr. ars.

— **lying** down at night and in sleep cough is absent: Kali b.[6]

— — — a hacking dry cough from tickling in larynx, or dryness in pharynx, worse at night as soon as he lies down: Phyto.[7]

— Compare with Lying.

— **moves**, night cough worse if she raises herself or moves; wakes

**NIGHT**—(*Continued*).
her about 3 A. M. with headache: Nitrum.
— **paroxysmal** cough: Arg. n., Aur. mur., Aur. s., Carbo v., Hepar, Merc. c.
— — — every other night on falling asleep: Merc. s.
— **periodic** cough, quartan, about 12 or 2 at night: Cocc.
— **racking** cough: Chin. s.
— — — every other night, on falling asleep: Merc. s.
— racking cough at, with frequent arrest of breathing, stitches in chest, sore throat and fever: Nitr. ac.[6]
— **raises**, night cough worse if she raises herself or moves; wakes her about 3 A. M. with headache: Nitrum.
— **rough** cough: Lyc.
— **scraping** cough: Natr. m., Rhod.
— — — on waking: Calc.
— **shaking** cough: Anac.
— **short** cough: Bell.
— — — agg. at: Codein., Coloc.
— — — in bed: Arg. n.
— **sleep**, incessant coughing prevents: SEPIA.
— — cough at, prevents sleep and causes exhaustion: PULS.
— — in bed, cough after having slept an hour or two, occurring before midnight, wakes patient: *Aralia*.
— **stomach**, nightly cough affecting stomach and diaphragm, mostly before sunset: LYC.
— **sudden** cough: Apoc. c.
— **suffocative** cough: Ars., Bell.
— — — like whooping cough, with intense pain: China.[6]
— **tickling** cough: Arg. n., *Coloc.*, Natr. m.
— — — agg. at: Zinc.

**NIGHT**, **violent** cough from tickling in larynx: *Cycl.*
— — — inducing vomiting, or a suffocative cough before and after going to bed, also at night: Indigo.[6]
— **violent** racking cough every other evening, when on the point of going to sleep, as if chest and head would fly to pieces, for a half hour; the cough is followed by violent stretching: Merc. s.[6]
— very violent cough, succeeded by violent hoarseness, with chilliness from morning till evening: Cupr.[6]
— violent cough for many nights, with irritation, as if from stomach, both while waking and during sleep: Merc. s.[6]
— violent cough during sleep with gnashing of the teeth: Bell.[6]
— **waking** from the cough: Caust.,[12] Cocc., Hepar, Kali c.,[2] Lach., Mag. m., Nitrum, Phos., Sang., SEP., Sil.,[1] Squil.,[7] Stront.,[12] SULPH.[1]—5.
— — at 2 A. M. from the cough: Cocc., Nitrum.—5.
— — 3 A. M., cough wakes at, with stupefying headache; worse if she raises herself or moves: Nitrum.
— — dry cough at, waking from sleep with intense pain in chest; little cough during day: *Kali c.*[6]
— — dry cough waking from sleep: SULPH.
— — frequent cough at, wakes him, after which he again falls to sleep; almost incessant cough while lying, disappears on sitting up: HYOS.
— — frequent cough waking her from sleep, with rattling in scrofulous subjects: Bell.[6]
— — from sneezing, with tickling in throat, causing cough: Aur. mur.[7]

**NIGHT**, See Sleep, Waking, etc.
— **whooping** cough at: Ambr., Anac., Ant. t., Arn., Ars., Bar., Bry., Carbo v., *Cham.*, China, Coc. c., Coral., Cupr., Dros., Dulc., Hepar, Hyos., Meph., Mez., Mur. ac., Natr. m., Nitr. ac.,[1] Puls., Samb., Seneg., Sep., Sil., Spong., Sulph., Sul ac., Verat.—2.

**NIPPLE**, cough with pain under left nipple through to scapula: Comocladia.

**NOISE** agg. cough: Arn., Ph. ac.—5.

**NOISELESS** cough: Calad., Dros.—5.

**NOISY** cough: *Bell.*

**NOON**, cough at: Agar., Arundo, Bell., Staph.
— — from fullness of chest: Sulph.
— — in sleep: Euphr.
— **deep** cough toward noon, with discharge of pure blood: Sil.
— — — amel. noon and lying down: MANG.
— **dry** cough at: Sulph.
— — — from tickling in palate and pharynx: Arg. n.
— — — — — at top of throat: Naja.
— **exhausting** cough at, from tickling in palate and pharynx: Arg. n.
— **hacking** cough at, from tickling in palate and pharynx: Arg. n.
— — — from tickling at top of throat: Naja.
— **hollow** cough toward: Sil.
— **suffocative** at: *Arg. n.*
— **violent** cough about, several days in succession, raising a large quantity of tenacious mucus: Bell.[6]

**NOSE**, bleeding of, with cough: Acon., Arn., Bell., Bry., Carbo an., Carbo v., Cina, Cupr.,[5] DROS., Dulc., Ferr., Hyos., Indigo,[5] Iod., Ipec., Kreos., Led., Merc., Mosch., Mur. ac., Natr. m., Nitr. ac., Nux v., Phos., Puls., Rhus, Sabad., Sep., Sil., Spong., Sulph., Sul. ac.—2.

**NOSE**—(*Continued*).
— **bleeding** at every cough: INDIGO.[5]
— — at night when coughing: Natr. m.[10]
— — of, and vomiting from violent cough: Indigo.[10]
— **blowing**, excites cough: Arn.[5]
— **burning** in, with cough: Cina, Mez., Sars., Sulph.—2.
— **coryza** with cough: Acon., All. c.,[5] Alum., Ambr., Ars., Bar., Bell., Calc., Canth., Carbo an., Caust., Cimex, Con., Dig., *Euphr.*, Gels.,[5] Graph., Ign., Kali c., Kali chl., Lach., Lyc., Mag. c., Meph., Merc., Natr. c., Nitr. ac., Nitrum, Phos., Ph. ac., Rhus, Rumex, Sang., Sep., Sil., Spig.,[6] Spong., Staph.,[6] *Sulph.*, Sul. ac., Thuja.—12.
— — after cough: Coca.
— — **alternating** with cough: Colch.,[5] Lach.[11]
— — with cough, rawness in **chest** and much expectoration: Sulph.[6]
— — with **dry** cough: Bell.,[10] Graph.,[10] Natr. m., Nitr. ac., Selen., Sulph.
— — with dry cough, in sleep: Sep.
— — — — — in daytime, with stitches in right side of abdomen and dry coryza: Sulph.[6]
— — — — — headache and stitches in throat: Nitr. ac.[10]
— — — — — hoarseness, dryness in throat, and coryza of clear mucus, with cough: Sulph.[6]
— — — — — with violent hunger and cough: Sul ac.[6]
— — and cough with swelling of sub-maxillary glands, pain in

**NOSE**—(*Continued*).
throat during deglutition, great chilliness: Sil.[6]
— **coryza, fluent** with cough, headache, lachrymation, thirst, want of appetite, trembling of hands, feverish; worse evenings and indoors, better in open air: All. c.
— — — toward evening with cough: Thuja.
— — **increases** as cough diminishes: Mag. s.[6]
— — — irritation to cough: Sep.
— **discharging** when coughing: Lach.
— — water when coughing: Sulph., Zinc.
— **dryness** of, with cough: Calc., Dros., Nux m.,[5] Sticta.—2.
— **forced** inspiration through excites cough: Sapon.
— **itching** of tip of, before coughing, and itching low in lungs, and extending up through trachea to nose: Iod.
— **motion** of alæ nasi: *Lyc.*, Phos.
— **pain**, jerking and swelling at base of, on coughing or sneezing: Nitr. ac.[20]
— **rubbing** of, in children: Lyc., Squil.

**NURSING**, whilst suckling the child, cough with expectoration: Ferr.

**OBSTINATE** cough. See Constant, Persistent.

**OCCIPUT**, **aching** in, on coughing: Alum.,[2] Sulph.[1]
— **bruised** feeling in, on coughing: Tarent.
— **pain** in, with cough: Anac., Carbo an., Ferr., Mag. c., Mosch., Sep.—2.
— — ulcerative pain in, when coughing: Sep.,[6] Sulph.[6]
— See also under Head.

**ODORS**, strong, excite cough: Phos.

**OFFENSIVE** cough: Caps.[16]

**OLD** men, cough in: Con.[5]

**ŒSOPHAGUS**, **aching** in, from coughing: Ferr.
— **soreness** in, from coughing: Ferr.
— **tickling** in, excites cough: Arn.[5]
— — at bottom of, causes cough: Raph.[1]

**OPEN air.** See Air.

**OPPRESSION** of chest. See under Chest.

**OPPRESSIVE** cough: Ail., Phal.

**OVERHEATING**, cough from: Nux m., *Thuja.*[12]

**OVERPOWERING** cough, as if larynx were tickled by a feather, in evening before sleep: LYC.

**PAIN.** See Chest, Abdomen, etc.
— **inability** to cough, on account of the: Natr. slfc.
— — — — from pain in side: Ox. ac.[1]
— in **various** parts (as knee) from the cough: Caps., Guare., Lach.

**PAINFUL** cough: *Agar.*, *Ail.*, Ant. ox., Ant. t., Caps., Coc. c., Cop., Cori. r., Kali hypermang., Lact., Merc. c., Natr. c.,[12] Rhus,[12] Tarent., Ustil.—1.
— — **chest**, from irritation in lower: Kreos.
— — **evening** in bed: Bry.
— — **midnight**, before, waking: *Rhus.*
— — **night**: Caust.

**PAINLESS** cough: Stram.[5]

**PALATE**, cough, as if from an **acrid fluid** running over the: Kali b.
— **burning** in, causing irritation to cough: Acon. f.
— — up to arch of, cough from: Dig.

**PALATE, dryness**, roughness and scraping in soft, causing hacking cough: *Dros.*
— **irritation** from elongated uvula, cough from: Alum.,[8] Brom.[1]
— — — — —, worse mornings, dry cough: Brom.
— **stitches** in, causing inclination to, but no cough: Ph. ac.
— **tickling** on, excites cough: Nux v.,[5] Rei.[1]
— — — dry hacking cough: Tilia.
— — — dry cough, after lying: Carbn. s.
— — — hard palate and pharynx, excites a violent, laborious whooping cough, worse after midnight and in early morning: Nux v.[2]
— — — and pharynx, dry hacking or exhausting cough, noon: Arg. n.
— — — roof of, dry cough: Nux v.
— — — soft, dry cough: Fran.
**PALPITATION** of heart with cough: Arn., Calc., Kali b., Nitrum, Puls., Spong., Stram.—5.
— — — agg. by cough: Ib.
**PANTING** cough: Mur. ac.,[12] Sul. ac.[6]
— — with concussion in head: Rhus.[6]
— — preventing sleep: Calad.[7]
— — like whooping cough, worse on deep inspiration: Dulc.[7]
**PAROXYSMAL** cough: Acon., *Agar.*, Alum., *Ambra*, Ang.,[12] Aqu. petr., Arum. i., BELL., Bry., Calad., Cann. s., CARBO v., Carbn. h., *Caust.*, *Chel.*, China, Cimex, CINA, *Coc. c.*, Coff., *Con.*, *Coral.*,[12] Croc., *Cupr.*, Delph., Dign., DROS., Ferr., Ferr. mur., Gins., HEPAR, Hydro. ac., HYOS., Ign.,[11] Jatro., Kali cl., Kreos., Lact., Laur., Lina., Lobel.,[12]

**PAROXYSMAL**—(*Continued*).
Lyc., Mag. c., Mag. m., Mang., Merc., Merc. c., Merc. i. r., Morph., Natr. m., Nicco., Nitr. ac., Nitrum, Nux v., Op., Phos., Plumb., *Puls.*, RUMEX, Sabad., Sep., Sil., STANN., Sulph., Sul. ac., Tarent., VERAT., Zinc.—1 and 11.
— — **agg.**, afternoon, entering warm room: Anth. n.
— — — after chill: Phos.
— — — in doors: Chel.
— — — by eating warm food, amel. rinsing mouth with cold liquids or by cold drinks: Coc. c.
— — **amel.** before midnight by swallowing mucus: Apis.
— — — hard spells of cough not ceasing till masses of offensive sputa are raised: Carbo v.
— — — on sitting up: Cinnab.
— — — laying hand pit of stomach: *Croc.*
— — **afternoon**, tickling at back of larynx: Anth. n.
— — — agg. in, entering warm room: Anth. n.
— — **attacks**, first the strongest, the following one weaker and weaker: *Ant. cr.*[2]
— — — follow one another quickly: Ant. t., Cina, DROS., Hepar, Ipec., Merc., Sep., Sulph.—5.
— — — follow one another so as to scarcely permit respiration in the intervals: *Dros.*[5]
— — in **bed**: Cinnab.
— — — — evening, from tickling in back of larynx: Coc. c.
— — — — — salivation: Natr. m.
— — — —, on getting warm in: Naja.
— — — — before going to, night: Coc. c.
— — — — morning, from tickling in throat: Coc. c.

**PAROXYSMAL** cough, **bread** or cakes, from eating: Nitrum.
— — **breath**, cough commences with gasping for breath: Ant. t.,[7] Coral.[2]
— — after **chill**, agg.: Phos.
— — **chest**, from cramps in: Bell.
— — **commencing** with gasping for breath: Ant. t.,[7] Coral.[2]
— — **consisting** of but few coughs: Bell., Calc., Laur.—2.
— — — — long coughs: Ambra, Carbo v., CUPR., Lobel.—5.
— — — — short coughs: Alum., Ant. t., Asaf., Bell.,[5] Calc., Carbo v., Dros.,[5] Kali b., Lact., Squil.[5] —2.
— — — — three coughs: Stann.[2]
— — — — two coughs: Cocc., Laur., Merc., Puls., Sulph.—5.
— — **cry**, sudden loud cry and yawning, following paroxysms of dry cough: Opium.[6]
— — **day** in the: Nitr. ac.
— — after **dinner**: Aeth., Phos.
— — **dry**, tickling, paroxysmal cough, with gaping, worse at night, drowsy but cannot sleep; or with spasm of lungs and blue face: Opium.[7]
— — **eating** bread or cakes, from: Nitrum.
— — **evening**: Chel., Chlor., *Hepar*, Natr. m.
— — — at 1 or 2 P. M.: Aqu. petr.
— — — — 1.30 P. M.: Phel.
— — — — 2 P. M.: Ol. an.
— — — — 3 P. M.: Phel.
— — — — 4 P. M.: *Chel.*
— — — — 4 P. M., walking in hot sun: Coca.
— — — — 5 P. M.: Cupr.
— — — — 6.15 P. M.: Ol. an.
— — — — 7 P. M.: Grat.
— — — — 11 P. M.: after lying down: RUMEX.

**PAROXYSMAL** cough, **evening**, in bed, with salivation: Natr. m.
— — — from irritation in larynx, in bed: Cocc.
— — — after lying down: *Nux v.*
— — — — — — —, at 11 P. M.: RUMEX.
— — — salivation, in bed: Natr. m.
— — — in cool wind: Coca.
— — — walking in hot sun, 4 P. M.: Coca.
— — **forenoon**: Coc. c., Grat.
— — **frequent** paroxysms of dry, violent, hollow, or short, exhausting cough, excited by tickling in larynx which brings tears to the eyes: Chel.[2]
— — with **gagging**, worse after midnight: *Dros.*
— — **hard** spells of cough, not ceasing till masses of offensive sputa are raised: Carbo v. (Raue.)
— — **indoors**, agg.: Chel.
— — **isolated**, cough appears in isolated paroxysms and is very violent, ends with frequent sneezing: Agar.
— — **midnight**, about: *Cham.*, Naja.
— — — before, from crawling in windpipe near supra-sternal fossa: Apis.
— — **larynx**, from crawling in: *Psor.*
— — — irritation in back of, evening, in bed: Cocc.
— — — stitches in: Indigo.
— — — tickling in: Chel.,[2] Coc. c., Cycl., Lyc.
— — — — at back of, afternoon: Anth. n.
— — — — after dinner: Calc. f.
— — **lying** down, evening after: *Nux v.*
— — — — 11 P. M., after: RUMEX.

**PAROXYSMAL** cough, **morning**: Agar., *Ant. cr.*, Coc. c., Nux v., Ol. jec., Sang., Sul. ac.
— — — at 2 and 3.30 A. M.: Coc. c.
— — — — 10 A. M.: Aqu. petr.
— — — in bed, from tickling in throat: Coc. c.
— — — after waking: RUMEX.
— — **night**: Arg. n., Aur. m., Aur. s., Carbo v., Hepar, Merc. c.
— — — before going to bed: *Coc. c.*
— — — every other, on falling asleep: Merc. s.
— — — warm in bed: Naja.
— — **occurring** occasionally: Bad.[12]
— — — in rapid succession: Ant. t., Dros., Hepar, Ipec., Sep., Sulph.—12.
— — **salivation**, with, in bed, evening: Natr. m.
— — **sitting** up, amel.: Cinnab.
— — while **smoking**: All. sat.
— — compare with Spasmodic Cough.
— — **stomach**, laying hand on pit of amel.: *Croc.*
— — **sun**, walking in hot: Coca.
— — **suffocation**, suddenly, on swallowing: BROM.
— — **throat**, from mucus in: Atro.
— — — — rawness in: Coc. c.
— — — — scraping in: Coc. c.
— — — — tickling in: Caust.
— — **yawning** and sudden loud cry, following paroxysms of dry cough: Opium.[6]
— — **violent** paroxysms of cough, or frequent cough excited by tickling irritation which begins in larynx and extends down into trachea, followed by dryness of mouth and larynx: Hydro. ac.[2]
— — **waking**, morning after: RUMEX.
— — **walking** in cool wind, evening: Coca.

**PAROXYSMAL** cough, **walking** in hot sun, 4 P. M.: Coca.
— — **warm** in bed, night: Naja.
— — — room, entering, afternoon, agg.: Anth. n.
— — compare with Whooping Cough.
— — **windpipe**, crawling in, near supra-sternal fossa, before midnight: Apis.
— —**wind**, from walking in cool, evening: Coca.

**PARTURITION**, cough after: Rhus.[5]

**PECTORAL** muscles, stitches in, on coughing: Dros.
— — See Chest.

**PENIS**, darting in, as from congestion, on coughing: Ign.[6]
— erection of, while coughing: Cann. s.,[1] Canth.[2]

**PEPPER**, cough from eating: Cina.[2]

**PERIODIC** cough: Ars., Cocc., Coc. c., Colch.,[1] Lach., *Nux v.*—12.
— — barking, painless, shrieking tone, without expectoration: Stram.
— — **day** in the: Anac.
— — — every other: Nux v.
— — **hour**, every half, after midnight: Acon.
— — — at same every day: Lyc., Sabad.[10]
— — quartan, about 12 or 2 at night: Cocc.
— — speaking or smoking, from: Atro.

**PERSISTENT** cough: Acon., Am. caus.,[1] Bell., Cub., CUPR., Dios., *Hyos.*, Ipec., Jatro., Lyc.,[1] Merc., Mez., Nitrum, Rumex, Sang., Squil.—5.
— — See Continuous.
— — midnight, lying on back, amel. lying on side: *Nux v.*

**PERSONS**, other, coming into room agg. cough: Phos.[8]

**PERSPIRATION agg.** by coughing: Acon., Ant. t., ARS., Bell., Bry., Calc., Caps., Carbo v., China, Dig., *Dros.*, HEPAR, *Ipec.*, Kali c., Lyc., Natr. c., Natr. m., *Nitrum*, *Nux v.*, PHOS., Ph. ac., *Rhus*, *Sabad.*, Samb., Selen., SEPIA, *Spong.*, Sulph., *Verat.*—3.

— cough and expectoration with: Ant. t., ARS., Bell., *Bry.*, *Calc.*, *Dig.*, *Dros.*, Ferr., *Merc.*, *Natr. c.*, Nitrum, PHOS., Ph. ac., Puls., SEPIA, Sil., *Spong.*, *Sulph.*, *Thuja*, Verat.—3.

— **dry** cough with: Acon., Ant. t., Apis, Arg. n.,[5] ARS., Bell., Bry., Carbo an.,[5] *Caust.*, CHAM., Cimex, Coff., Con., *Dros.*, Dig.,[5] Eupion, *Hepar*, Hyos., Ign., *Ipec.*, Led., Lyc., *Merc.*, Mur. ac., Natr. c.,[5] Nitrum, Nitr. ac., *Nux v.*, PHOS., Puls., RHUS, Sabad., *Samb.*, SEPIA, *Spong.*, Stront., SULPH., *Verat.*—3.

— cough **agg.** after: Sil.[5]

— on **forehead** with cough: Ant. t., Ipec.—5.

— — — — — cold: Verat.[5]

— on **head**, with cough: *Ant. t.*, Calc., Ipec.,[12] Sil., Tarent.[1]—5.

— **inclination** to: China, Psor.—5.

— at **night**, with cough: China, Lyc., Natr. c., Psor.—5.

— **paroxysms** of cough end with: Ars., Brom.—2.

**PHARYNX, burning** in, excites cough: Ars., Caust., Ph. ac.—5.

— — and soreness in, from cough: Sulph.[6]

— **crawling** in, excites cough: Prun. sp.

— **dryness** in, excites cough: Caust.,[6] Cycl.,[5] Coc. c., Phyto.—1.

**PHARYNX, dryness**, a hacking dry cough from tickling in larynx or dryness in pharynx, worse at night, as soon as he lies down: Phyto.[7]

— — dry cough: Atro.

— — in morning, cough from: Phyto.

— on **inspiration**, tickling in, short cough: Olean.

— **irritation** in, agg. cough: Croc.[5]

— — — hacking cough: Trif. p.

— **paralytic** sensation in, with cough: Meng.[5]

— **pressure** in anterior wall of, cough from: Tarax.

— **scraping** at back of, painful cough from: Kali b.

— — in, causes cough: Arg. n.,[1] Cycl.[5]

— **sore** feeling in, from coughing: Caust.,[5] Sulph.[6]

— **tickling** in, excites cough: Anac., Ars., Cham., Coca, Mag. s., Olean., Sil.

— — from air passages to, hacking cough: Hydro. ac.

— — at back part of, excites cough: Coca.

— — of, and palate causes exhausting cough, noon: Arg. n.

— — hacking cough: Mag. s.

— — — — night: *Sil.*

— — high up in, dry or convulsive cough, evening, in bed, 10.30 P. M.: Carbn. s.

— — on inspiration, short cough: Olean.

— — night, hacking cough: *Sil.*

— — ungovernable, spasmodic cough, which seems as if it would burst the thorax, excited by a peculiar tickling in pharynx, which, again, is induced by a sensation of smothering in throat: Lact. v.[2]

**PHARYNX**, **tickling**, violent convulsive cough, agg. night and after eating; after the attack yawning and sleepiness: Anac.[10]

**PHARYNGEAL** cough: Sol. t. æ.

— — as when thrusting finger into back of mouth: Merc. c.

**PIANO**, cough when playing: Ambr., *Calc.*, Cham., Kali c., Kreos., Ph. ac.—12.

**POSITION**, cough agg. by being long in same: Coc. c.,[2] Ph. ac.[12]

**PRÆCORDIAL** region, dry cough from tickling at: Bar.

**PREGNANCY**, cough during: Calc.,[12] Con., Ipec., *Nux m.*, Phos.,[12] Puls., Sabin., Sep.—5.

**PRESSURE** on chest relieves pains from the cough: ARN.,[10] Bor., *Bry*, Cimic.,[6] *Dros.*, Eup. per., Kreos., Merc., Natr. m.,[10] Natr. sul., Phos., *Sep.*,[10] Ran. b. —5.

— on pit of stomach amel.: *Croc.*, Dros., Phos.

**PROVOCATION** to cough. See Irritation.

**PROSTRATION** with cough: ARS., Carbo v., Coral., Lach., Phos.,[1] Stram., Verat.—5.

— and dry cough, morning: Hyper.[7]

**PTYALISM** with cough: Ambr.,[5] Merc., Mez., Staph.,[5] Verat.—5.

**PYROSIS** with cough: Ambr., Amm. m., Ars., Bry., Mez., Spig., Staph.—5.

**PUFFING** cough, on taking deep breath: Naja.

**RACKING** cough: Anac., Amm. m., Ang., Ars., Amm. t., Aur., Brom., Calc., *Carbo v.*, Cann. i., Caust., Chel., China, Coc. c., Cocc., Con., Croc., Cupr., Daph., *Dulc.*, Graph., Gymn., *Hyos.*, *Ign.*, Ipec.,[5] Kali c., Lach.,[5] Lact., Led., Lyc., Mag. m., MERC., Merc. c., *Mez.*, Mur. ac.,

**RACKING**—(*Continued*).

Natr. c., NUX v., Op., Osm., Phos., PULS., Rhod., Rhus, Selen., Seneg., *Sil.*, Spig., Squil., Staph., Stann., SULPH., *Verat.*, Zinc.—1 and 11.

— — **chest**, from irritation in lower: Kreos.

— — **dry** racking cough, from tickling in larynx, lasting ten minutes; both dryness and cough relieved by a drink of water; they, however, returned in fifteen minutes, with so great violence that tears came into eyes, again amel. by water: *Opium.*

— — **evening**: *Ipec.*

— — — 10 P. M.: Natr. m.

— — on deep **inspiration**: Con.

— — **larynx**, from tickling in, amel. by drinking water: *Opium.*

— — **morning**, agg. in: *Chel.*

— — — on waking: Caust.

— — — much cough, especially in morning; cough racking, as in consumption, with much expectoration coming deep from lungs; the cough could not be prevented by changing the position, after waking, since the rattling in the chest increased; expectoration in large quantities, difficult to loosen; during the first days the paroxysms of cough, with pain behind the sternum, occurred especially at night: *Chelid.*

— — **night** at: Chin. s.

— — — every other, on falling asleep: Merc. s.

— — on **waking**, morning: Caust.

**RASPING** cough: Calc. s.

— — from dryness of throat: Stram.

**RATTLING** cough: *Ant. t.*,[11] Ang., Arg. n.,[1] *Bell.*, Bry., Calc., Caust., *Cham.*,[11] Con.,[11] Hepar, IPEC., Kali b., Lyc., Meph., Merc. c.,[1]

**RATTLING**—(*Continued*). Natr. c., *Natr. m.*,[11] *Nux v.*, Puls., Samb., Sul. ac., Yuc.[1]—5.
— — in **day** and in room, not at night or in air: *Arg.*
— — when **eating**: *Phos.*
**RAW** cough: Natr. m.
**READING** agg. cough: Cina, Mang., Ment. pi.,[1] Meph., *Nux v.*, Phos.—12.
— See Mental Exertion.
— **aloud**, agg. cough: Ambra, Mang., Meph., Nitr. ac., Par., Phos., *Stann.*, Verb.—5.
— — causes dry cough: *Mang.*
— — — — — in evening: *Phos.*
— — **irritation** to cough from: Nitr. ac.
**RECTUM**, stitches in, on coughing: Nitr. ac.[5]
— See also Hæmorrhoids.
**RE ECHO**, cough seems to re-echo in stomach: Cupr. (E. W. B.)
**REFLECTION** agg. cough: Arn., Asar., Cist., [10] Cocc., Ign., *Nux v.* —5.
**REPOSE**. See Resting.
**RESPIRATION** (breathing) agg. cough: Amm. c., Asar., Bell., Coloc., Dulc., Graph., Hepar, Ipec., Mag. m., Natr. m., Nitrum, Sulph.—5.
— **arrested**, continued dry hacking cough, with vomiting and arrest of breathing: ALUM.
— — at night during cough: Natr. m.
— — when coughing: Euphr., Led., Merc., *Sepia.*
— — See Interrupted.
— **deep**, agg. cough: Acon., Amm. c., Amm. m.,[1] Arn., Ars.,[1] Bell., Bism., Brom., Bry., Carbo an., China, Cina, Coc. c., Con., Cupr., Dros., Dulc., Euphr., Ferr.,[2] Graph., Hepar, Ipec., Kali c., Lyc., Mag. c.,[2] Mag. m., Meny.,

**RESPIRATION**—(*Continued*). Mez., Mur. ac., Natr. m., Nitr. ac., Ph. ac.,[2] Puls., Rhus, Sabad., Samb., Seneg., Sep., Sil., *Squil.*, Stann., Stram., Sulph., Verb., Zinc.—2 and 11.
— **deep**, irritation to cough on: Cina, *Lyc.*
— — causes short cough: Naja.
— **deficient**, agg. cough: *Aur.*,[1] Cina,[6] Cocc., Coloc.,[1] Dros., Euphr., Guai., Hepar, Ign., Ipec., Lyc., Nux v., Spig.—5.
— — with cough: *Ars.*, Ferr., *Ipec.*, Op.—5.
— **difficult** after coughing: *Ars.*,[5] Phos.[7]
— — — desire to cough. Mosch.[5]
— — with cough and violent bronchitis, at first without fever, with slow pulse, great prostration and emaciation: *Phos.*
— **every**, causes desire to cough: Ipec.[5]
— **gasping**, with cough: Ant. t., Brom.—5.
— — for breath before attack of cough: Ant. t.,[7] *Brom.*,[5] Bry.,[6] Coc. c., Coral.
— — for breath, immediately before the cough, quick spasmodic inspirations as if child could not draw a full breath: Bry.[6]
— **holding** the breath excites cough: Nitrum, Sprun. sp.[1]
— **interrupted** (short, stoppage of, etc.), with cough: *Acon.*, *Alum.*, Amm. c., Anac., ANT. T., Aralia, Arg. n., Arn., ARS., Bar., *Bell.*, Brom., Bry., Calad., Calc., Carbo an., Carbo v.,[11] Caust., CINA., Cocc., Clem., Con., CUPR., Dolich.,[11] DROS., Euphr., Ferr.,[5] Guai.,[6] Hepar,[11] Ign.,[12] Iod.,[6] IPEC., Kali b., Kali chl.,[6] *Kreos.*, Lach., Led., Lyc., Merc., Mosch., Natr. m., Natr. s.,[12] Nicc.,[12]

**RESPIRATION**—(*Continued*). Nitr. ac., Nitrum, *Nux m.*, NUX v., OP., Phell.,[12] Phos., Puls.,[12] Rhus,[6] Sep.,[1] *Sil.*, Spig., Squil.,[6] Spong., Verat.,[12] Zinc.,[12] Zing.[10] —5 and 11.

— **interrupted**, with desire to cough, blue face, then falls into deep sleep with cold sweat: Opium.[5]

— — almost suppressed from coughing: CUPR.

— — irritation to cough is so sudden and violent one can scarcely inhale at all: *Sepia.*[7]

— **interruption** of, in upper trachea, evening, causes cough: Ign.

— **short**, after coughing: *Ars.*,[5] PHOS.[1]

— **short**, before cough: Ant. t.,[7] Ars.,[6] Bry., Caust.,[1] Coc. c., Coral., Led., Lyc.—5.

— — before cough, afterward dizziness: Led.[7]

— — can scarcely breathe for coughing: *Dros.*, *Euphr.*, Led., Merc., Sepia.

— — from painful, anxious, short cough, that wakes her before midnight: *Rhus.*

— — cough as though from: Euph.

— — from rapid turns of coughing: Dros.

— — from single coughs of ringing sound, which follow each so quickly, worse after midnight: *Dros.*[17]

— — from titillating cough, worse at night: Zinc.[6]

— **sighing** with cough: Caps.[5]

— **snorting** with cough: China.[6]

— **stertorous** with cough: Bell.[5]

— **suffocative**, arrest of, and opisthotonus, previous to cough: Led.[10]

**RESPIRATION**, **taking** deep, causes puffing cough: Naja.

— **tightness** of breathing, causes suffocative cough: *Hepar.*

— **want** of. See Deficient.

— **wheezing** and panting, before the cough: Kali c.[2]

**REST** agg. cough: Ambra, Ars., Caps., Coc. c., Dros., Dulc., Euph., Euphr., Ferr., Hyos., Mag. c., Mag. m., Nux v., Phos., Ph. ac., Puls., Rhus r., Rhus, Sabad., Samb., Seneg., Sep., Sil., Stann., Sulph., Verb., Zinc.—5.

— **amel.** cough: Lach., Psor., Puls. —5.

— long in one position agg. cough: Coc. c.,[2] Ph ac.[12]

**RESTLESSNESS** with cough: Acon., Ars., Coff., Iod., Lach., Samb.—5.

**RETCHING** cough: Natr. m.

— with cough: Bell., Brom., CARBO v., *China*, DROS., HEPAR, Ipec., Kali c., KREOS., Lyc., Merc., Mez., Natr. m., NUX V., *Puls.*, Sep., Squil., Stann., SULPH.—11.

**REVELING**, agg. of cough after: Stram.[5]

**RIB**, pain in last short, on coughing: Meph.[5]

— scratching beneath right fourth or fifth, excites dry cough: *Acon.*

**RIBS**, bruised feeling in, on coughing: Arn.[5]

— pressure below short, on coughing: Spong.

**RIDING** agg. cough: Sul. ac.

**RIGIDITY** of body with cough: Bell., Cina, *Cupr.*, IPEC., Led., Mosch.—2.

— — — before cough: Cina.[10]

**RINGING** (clear) cough: Acon. All. c., Ars., Dros., Stram.—12.

— croupy cough with ringing sound: Apis.[7]

**RINGING** cough, from irritation of throat caused by hot eructations, amel. by smoking: Lac. ac.

**RINSING**, voice hoarse and rough, with irritation to cough, slight paroxysms of cough, and hawking of mucus, relieved by rinsing mouth with tepid water or eating warm food every morning: Coc. c.

— awoke at 6 A. M., cough intermitting for several minutes, at first barking, clear, and dry, afterward becoming looser, with expectoration of some tenacious mucus, the hawking of which provoked vomiting several times; with sensation of soreness in throat and pressive frontal headache; cough relieved on rinsing mouth with cold water and drinking a few swallows, which was very refreshing to the sensation of heat in throat; this latter, however, returned after a half cup of warm milk, but was not so severe as before; cough worse in hot room: *Coc. c.*

**RISE**, frequent hacking cough, evening when in bed, with bitter taste in throat till he falls asleep, and in the morning similar cough and taste, lasting till he rises: *Rhus.*

— dry cough, at night, waking, must rise up: Mag. m.

**RISING**, **after**, cough agg.: Acon., Alum., Bar., Carbo an., Ign., Mag. c., Natr. s., Phos., Sep., Sul. ac., Thuja, Verat.—2 and 5.

— — cough amel.: Mag. c., Rhus.

— — — — compare with Sitting up.

— — **hacking** cough: Benz. ac.

— — — — amel.: Rhus.

— — **morning**, cough in: Euph., Lach., Nitr. ac., Staph., Thuja.

**RISING**, **after morning**, cough from tickling in throat: Alumen.

— — — cough with expectoration of transparent mucus, and torn sensation in middle of sternum: *Phos.*

— — — choking cough: CINA.

— — — dry cough: Alum., Ang., Bar., Bov., Carbo an., Dig., Natr. slfc.

— — — gagging cough: Cina.[11]

— — — hacking cough: Arg., *Arn.*, Par.

— — — — — tickling in throat: Ox. ac.

— — — — — tickling low in trachea: *Arn.*

— — — hollow cough: Cina.

— — — irritable cough: Arg.

— — — irritation to cough: Alumen.

— — — and night, mucous cough: Sulph.

— — — short cough: Arn.

— — — cough when rising from stooping in: Phos.

— **before**, violent cough and expectoration of clotted blood, with sore chest in morning: NUX V.

— **on**, cough: *Ars.*, Chin. s., *Ferr.*

— **on**, from bed, cough: Acon., Bry., Calc. s.,[1] Carbo v., Chel.,[1] Cocc., Con., Lach., Mag. c., Natr. s., Phos., Sep., Tarent.,[1] Thuja, Verat.—5.

— on rising from recumbent posture, cough: Lach.[11]

— — **morning**, cough: *Ars.*, Chin. s., FERR., Hal., Indigo, Sil.

— — — cough, and continues until lying down again, can scarcely get breath; none at night, worse from smoking, better from eating: *Euphr.*[7]

— — — dry cough: *Carbo an.*, Grat.

**RISING**, **on**, violent fits of coughing before retiring and on rising: Ailan.[7]

— — — See Rising from Bed.

— — — coughs up blood: *Ferr.*

— — compare with Sitting up.

**ROOM**, cough **agg.** in: Arg., Bry., Croc., Laur., Mag. c., Mag. m., Natr. m., Puls., Spig.—5.

— **damp**, spasmodic cough, worse in damp room: Bry.[2]

— **entering** cold room from a warm, agg. cough: *Carbo v.*, Phos., *Rumex.*[7]—5.

— — nervous cough, when any one enters the room: Phos.[7]

— **warm**, agg. cough: Ambra, Arn., Ars., Bry., *Coc. c.*, Dig., Dros., Ipec., Laur., Lyc., Mez., *Natr. c.*, Puls., Seneg., Spong., Verat.—5.

— — cough in; amel. in cold room: Coc. c.[10]

— — entering a warm room from open air, agg. cough: Acon.,[12] Ant. cr.,[7] Bov.,[1] Coc. c., Natr. c., Sep.,[1] Sulph.[1] (afternoon), Verat, —10.

— — — — — cold air, cough from tickling in chest: Bov.

— — entering warm room, tickling in trachea, evening, dry cough: Com.

— — — —, afternoon, agg. dry cough: Anth. n., *Natr. c.*

— — — —, afternoon and evening, agg. violent cough: *Natr. c.*

— — on entering warm room from cold air, feels sensation in trachea as if full of smoke, which excites cough; feels as if he could not inhale sufficient air: Bry.[6]

— — going from warm room to open air, excites cough: *Acon.*

— **warm room**, going from, to cold air, or *vice versa*, causes coughing: Sepia, Nux v.,[10] Natr. c.[10]

**ROUGH** cough: Acon., Bell.,[2] Brom., Cann. ind., Carbo an., Carbo v., Card. b., Cop., Eupat. per., Eupion, *Hepar*, Mag. m., Meli., Natr. c., *Rhus v.*, Sep., Tarent., Ust.—1.

— — *agg.* going from cold air to warm room, or *vice versa:* Sepia.

— — *amel.* in open sir: Iod.

— — — sitting upright: Natr. c.

— — **chest**, from sticking and pressure in: Iod.

— hoarse, hacking cough, excited by sensation of soreness and heat in bronchi, worse in evening: Eup. per.[2]

— cough, **midnight**: *Nitr. ac.*

— — at **night**: Lyc.

— — **throat**, from mucus in: Kreos.

— — — roughness and crawling in: *Carbo v.*

**RUBS**, child constantly rubs eyes, nose, and face with his fist during coughing spell: *Squil.*[14]

— child starts out of sleep rubbing its nose: Lyc.[7]

**RUNNING**, agg. cough: Cina, Iod., Merc., Seneg., Sil., Stann., Sul. ac.—2.

**SACRAL** region, pain in, with cough: Amm. c., Merc., Nitr. ac., Sulph.—5.

**SALIVA**, flow of, agg. cough: Valer.[5]

— watery during cough: Cycl., Lach.

— water in mouth with cough: Ambr., Ars., Bry.,[16] Lach., Staph. —6.

**SALIVATION**, evening in bed, excites paroxysmal cough: Natr. m.

— with cough: Verat.[12]

**SALT** food agg. coughing: *Con.*, Lach.—2.

**SCABS** are coughed up every few weeks: Ferr.[7]

**SCALP**, perspiration on, on coughing: Ant. t., Tarent.

— — See Head.

**SCAPULA**, pain between or in region of scapulæ, worse from riding, sneezing, gaping or coughing, or any jarring: Calc.[7]

— cough with pain under left mamma through to left scapula: Como.[14]

— soreness between, with cough: Sul. ac.[7]

— Compare with Shoulder.

**SCARLATINA**, cough following: Ant. c., Con., Hyos.—2.

**SCRAPING** (rough) cough: Bell., Cham., Cimex, Coff., Dros., Eupat. per., Eupion, Grat., *Hepar*, Kali c., Kreos., Merc., Natr. c., Nicc., Nitr. ac., *Nux v.*, Plan., Puls., Sabad., Samb., Sep., Spong., STANN., Zing.—1 and 5.

— — with sore pain in whole chest, and alternating hoarseness, heat, and burning hands and soles; bruised feeling in legs, loss of appetite, nausea, heat, and perspiration at night, without thirst, and constipation: Natr. c.

— — dry cough: Kreos.[5]

— — **chest** sore; must support it with hands; flushed face; tearful eyes: Eup. per.[7]

— cough, evening: Rhod.

— — — after lying down: Bry.

— — night: Natr. m., Rhod.

— — — on waking: Calc.

— — from sticking in throat: Lyc.

**SCRATCHING** cough: Kali c.

**SCREAMING** (or weeping) excites cough: Ant. t., *Arn.*, *Cham.*, Verat.—5.

— and weeping with whooping cough: Stram.[8]

— with cough: Op., Samb.[5]

**SCREAMING.** See Crying.

**SCREECHING** shrill cough, in painless paroxysms: Stram.[2]

**SCROBICULUS** cordis. See Stomach, pit of, and Epigastrium.

**SCROFULOUS** children, chronic cough in, with swollen glands and enlarged tonsils; worse after slightest cold: Bar.[7]

**SENSES**, loses, from violence of the cough: Kali c.[6]

**SERIES** of cough: Phos., Sum.

— — — 10–11 A. M.: Sum.

— — — See Paroxysmal Cough.

**SEVERE** cough: Ipec., Cupr., Lobel., Sang., Staph.—5.

— continued cough, with circumscribed redness of cheeks: *Sang.*[5]

— — dry cough, mostly in the morning, with retching and desire to vomit, and sensation as if stomach were turned inside out: *Puls.*[10]

— long coughing spells: LOBEL.[5]

— cough, especially after lying and sleeping: Apis.[7]

— suffocative cough: IPEC.[5]

— uninterrupted cough, cannot speak a word, discharge of bloody mucus from nose, after sea wind: *Cupr.*[7]

— Compare with Spasmodic Cough.

**SIESTA**, cough during, from irritation in upper part of larynx: Arnic.

— short cough after, from irritation and tickling behind sternum: Rhus.

**SHAKE.** When coughing child seems to shake all over: Puls.[6]

**SHAKING** cough: Alumen, *Anac.*, Ant. cr., Arg. n., Arn., Ars.,[16] Bell., Bry., China, Hyos., Ign.,[16] *Ipec.*, Lach., Led., Lyc., Merc., Nitr. ac., Nux v., Olean., Phos., *Puls.*, Rhus, Seneg., Sil., Stann., Sulph., Sul. ac., Sum.—1 and 5.

**SHAKING** cough, **amel.** by discharge of mucus: Alumen.
— — — by sitting up: Arg. n.
— — from scratching in larynx: Alumen.
— — — tickling in larynx: Bry.
— — at night: Anac.
— (shattering) of the **body** by the cough: *Anac.*, Ant. c., Bell., Hyos., Ipec., Led., Olean., *Puls.*, Rhus, Senega, Stann.—2 and 5.

**SHARP** cough: Arn., Calc. s., Staph.
— — after eating: Staph.

**SHATTERING** cough: Ant. ox., *Rhus*, Sil., STANN.
— — from tickling itching in pit of throat: Sil.
— shattering, barking spasmodic cough, excited by a tickling in the larynx and in the epigastrium; worse evening until midnight, rarely only in daytime; expectoration morning and day: Nitr. ac.[2]
— shattering spasmodic cough, excited by tickling creeping in larynx, trachea, and chest; worse evening till midnight; no expectoration in evening: Rhus.[2]
— violent, hollow, shattering spasmodic cough, excited by tickling in larynx with suffocative arrest of breathing; worse evening till midnight; expectoration after midnight and in morning: Led.[2]
— shattering spasmodic whooping cough, often in paroxysms of two coughs each, excited by itching-scratching with a feeling of dryness and, as it were, of sulphur vapor in trachea and in chest; worse evening till midnight; expectoration morning and day: Puls.[2]
— shattering cough like whooping cough, excited by burning and

**SHATTERING**—(*Continued*).
tickling in larynx; worse evening and night; morning expectoration: Senega.[2]
— shattering spasmodic whooping cough, with frequent, rapidly succeeding coughs, excited by tickling as if from mucus firmly seated in trachea; expectoration in daytime, worse at night, especially after midnight: Hyos.[2]
— See also Shaking Cough.

**SHIVERINGS** with cough: Grat., Kreos.—16.

**SHOCKS**. See Paroxysmal Cough.

**SHORT** cough: ACON., *Aesc. h.*,[1] Aeth.,[2] Agar., *Alum.*, Anac., Ang., Ant. c., Ant. t., *Apoc. c.*, Arg., Arg. n., Arn., Ars., Asar., Aur., Aur. mur.,[1] *Bell.*, Berb., Brom., *Bry.*, Calc., Calc. s.,[1] Canth., Carb. ac.,[1] Carbo v., Carbn. ox.,[1] Casc., *Caust.*, Chel., *China*, Cimic., Cina, Coc. c., *Cod.*, COFF., Colch., Coloc., Con., Cop.,[16] Cupr., Dig., Dign., Dros., Dulc., Eupat. per., Euph., Eupion, *Graph.*,[16] Hepar, Hydro. ac., Hyos., Hyper.,[16] *Ign.*, Iod., Ipec., Jatro., Kali b., Kali c., Kali iod.,[12] Kali hypermang., Kobalt., Kreos., *Lach.*, Lachn., Lact., Laur., Linu.,[1] *Lyc.*,[16] Lobel., Marum, *Merc. s.*, Mur. ac., Naja, Natr. c., *Natr. m.*, Nitr. d. s.,[1] *Nitr. ac.*, Nitrum, *Nux v.*, Oena.,[1] Olean., Osm.,[1] Pau. p.,[1] *Petro.*, *Phos.*, Pin. s.,[1] *Plat.*, *Plumb.*, Podo., Puls., Rhus, Rumex a.,[1] Sabad., Senega, SEP., Sin. n.,[1] Spig., Spong., *Squil.*, STANN., Sticta, *Stil.*,[1] Stront., Sulph., *Sul. ac.*, Tabac.,[1] *Tellur.*, Teplitz,[1] Thuja, Zinc., Zing., Ziz.[1] —5 and 12.
— — **agg.** afternoon: Anac.
— — — evening: IGN., Phos.

**SHORT** cough, **agg.** open air: Senega.
— — agg. by coughing: IGN.
— — — after eating: Caust.
— — — lying after eating: Tereb.
— — — night: *Cod.*, Coloc.
— — — on swallowing: *Aesc. h.*
— — — walking fast: Senega.
— — **afternoon**: Chin. s.
— — — 2 P. M.: Laur.
— — — 5 P. M.: Natr. m.
— — — agg. in: Anac.
— — **air, open**: Spig.
— — — — agg. in: Senega.
— — — — walking in: Ang.
— — **air passages**, from tickling in: Ant. t.
— — in **bed**, evening: Lyc., *Sep.*
— — — — night: Arg. n.
— — **chest**, from hot constriction in: Carbo v.
— — during **coryza**: *Tellur.*
— — **coughing** agg.: IGN.
— — **day**: Arg., Cot., Kali b., Natr. c., Phos.
— — after **dinner**: Agar.
— — **dry** cough: CINA.[5]
— — after **eating**: Anac.
— — — — agg.: Caust.
— — — — agg. on lying: Tereb.
— — **evening**: Alum., Bar., Carbo v., Kali b., *Sep.*, Thuja.
— — — agg. in: IGN., Phos.
— — — in bed: Lyc., *Sep.*
— — — tickling in larynx: Cimic.
— — — from smoking: Thuja.
— — — from tickling behind upper sternum, 11 P. M.: Rhus.
— — — from interruption of respiration in upper trachea: IGN.
— — — on undressing: Chel.
— — **forenoon**: Agar., Alum.
— — — 7 A. M., on waking: Dign.
— — — 11 A. M., aching behind sternum on sitting bent forward: Rhus.

**SHORT** cough, **forenoon**, from irritation in trachea: Coc. c.
— — from **hawking**: Eugen.
— — short **hollow** unceasing cough, excited by creeping tickling, and much mucus in throat; worse evening till midnight: Bry., Caust.[2]
— — deep **inspiration**: Con.
— — **larynx**, from irritation in: Amm. c., Senega.
— — — — mucus in: Senega.
— — — — tickling in: Agar., *Ang.*, Graph., Iris v., Kali b., Laur., Led., Mez.
— — — — — — evening: Cimic.
— — — continued tickling in, 3 P. M., rather lessened on inspiration, followed by short, dry, shaking cough almost wholly ceasing on sitting up: Arg. n.
— — — from tickling in, only when walking in open air, with rattling in chest and expectoration of much yellow mucus: Ang.
— — — — sensation of thickening of the mucus membrane: Ery. a.
— — **lying**, after eating, agg.: Tereb.
— — **midnight**, after: Acon., *Ars.*
— — — before, waking patient: *Rhus.*
— — **morning**: Agar., Kali b., Lyc., Thuja.
— — — after rising: Arn.
— — — — tea drinking: Ars.
— — — from sensation of mucus in throat, after rising: Am. bro.
— — when **moving**: Carbn. ox.
— — **night**: Bell.
— — — in bed: Arg. n.
— — in **paroxysms**: Alum., Ant. t., Asaf., Bell., Calc., Carbo v., Dros., Kali b., Kali c., Lact., Squil.—12.
— — **pharynx**, from tickling in, on inspiration: Oleand.

**SHORT** cough, on deep **respiration**: Naja.
— — — from interruption of respiration in upper trachea: Ign.
— — **rising**, morning after: Arn.
— — — — sensation of mucus in throat, on: Am. bro.
— — after **siesta**, irritation and tickling behind: Rhus.
— — **sitting** up, amel.: *Arg. n.*, Natr. c.
— — when **smoking**: Coca.
— — — — evening: Thuja.
— — **sternum**, aching behind upper sternum, on sitting bent forward, 11 A. M.: Rhus.
— — — from irritation behind upper: Natr. ars., RHUS.
— — — irritation and tickling behind, after siesta: Rhus.
— — — from tickling behind upper: RHUS.
— — — — — beneath: Zinc.
— — **stomach**, on pressure of pit of: Tax.
— — on **talking**: Ant. t.
— — after **tea-drinking**, morning: Ars.
— — **throat**, from crawling in: Lach.
— — — — irritation in; Carb. ac.
— — — — mucus in: Senega.
— — — — sensation of mucus in, morning on rising: Am. bro.
— — — — rawness in: *Acon.*, Ol. an.
— — — — roughness in: Verat. v.
— — — — tickling in: Colocn., Iod., Mag. c.
— — **thyroid** cartilage, irritation to cough from tickling beneath: *Squil.*
— — — from tickling in region of: *Puls.*
— — **trachea**, irritation in, forenoon: Coc. c.
— — — — irritation in upper: Euph.

**SHORT** cough, **trachea**, from scraping in: Sabad.
— — — — sensation of something in: Sin. n.
— — — — tickling in upper: Mar.
— — on **undressing**, evening: Chelid.
— — on **waking**, 7 A. M.: Dign.
— — — patient, before midnight: *Rhus.*
— — **walking** fast agg.: Senega.
— — — in open air: Ang.

**SHOULDERS**, **pain** in, with the cough: Amm. c., Ars., China,[16] Bry., Dig.,[16] Lach., Phos., Puls., Thuja.—12.

— pain in left, with cough: Ferr.

— — between when coughing: Calc.,[5] Kali b.,[12] Stram.[1]

— **stitches** in, from coughing: Hyper.,[1] Puls.,[1] *Sep.*,[5] Merc.[5]

— stitches in left: Sulph.[5]

— — — right: Borax.[5]

**SHRILL** screeching cough, in painless paroxysms: Stram.[2]
— cough, on waking: Sol. t. æ.

**SHUDDERING** when coughing Puls.

**SIBILANT** and dry cough, like a saw driven through a pine board: *Spong.*
—, wheezing cough: Kreos., Prun. sp., *Spong.*—16.

**SIGHT** cloudy when coughing: Sulph.

**SIDE**, cough with crampy lumbago, left side: Chin. s.
— **lying** on, **agg.** or excites cough: Bar., Carbo an., Kali c., Kreos., Lyc., Merc., Phos., Puls., Seneg., Sepia, Stann., Sulph.—2.
— — — **amel.** dry, exhausting cough, about midnight; worse lying on back: *Nux v.*
— — — **left**, agg. or excites cough: Acon., Bar., Bry., Ipec.,

**SIDE, lying** on left—(*Continued*). Lyc., Merc., Paris,[1] *Phos.*, Puls., Rhus, Rumex, Senega, Sepia.—5.
— — — **painful**, agg. cough: Acon.[2]
— — — **right**, agg. or excites cough: Acon., Amm. m., Carbo an., Cina, Ipec., STANN.—5.
— — — — dry cough, night: *Carbo an.*
— — — — or back agg. irritable cough: Phos.
— — — — cough evenings, after lying down, sputum is loose and easier when turning from left to right side: Thuja.[7]

**SIDES, pain** in, with cough: ACON., Ambra, Amm. m., Ars., *Bell.*, Bor., BRY., China, Nitrum, *Phos.*, *Puls.*, Senega, SQUIL., Sulph., Verat., Zinc.[5]—11.
— pain in loins and sides, with cough causing him to hold them: Kali b.[6]
— pain in left side when coughing with weakness and dyspnœa: Verat.[6]
— pain in, causing inability to cough: Ox. ac.
— **stiches** in, with cough: Acon., Arn., *Bry.*, Phos., Squil., Zinc.[5]—12.
— — — preceding the cough: Zinc.[6]
— — — with troublesome cough, during chilly stage of fever, or after midnight: China.[6]
— — in right, lower: *Kali c.*[12]
— — left side: Natr. s., *Rumex*.
— See under Chest, and Abdomen.

**SINGING** agg. or excites cough: Dros., Meph., Phos., Spong., Stann., Stram.—5.
— on beginning to sing, hoarseness, weakness, and emptiness in chest, so that she was constantly obliged to stop and take a deep breath; at times a few expulsive coughs removed the hoarseness for a moment: STANN.[1]

**SIT up, must**: As soon as cough commences: Coc. c., Lach.—10.
— — — fatiguing dry cough, in morning after waking, obliging one to sit up, which relieves: Mag. s.[6]
— — — cough compels patient to sit up, it is moist and rattling, but no expectoration: *Ant. t.*[7]
— — — cough compels patient to spring up immediately and involuntarily: *Bry.*[7]
— — — evening cough directly after lying down, has to sit up: *Ars.*[7]
— — — evening while lying violent cough, must rise: Lith. c.[7]
— — — dry cough at night, wakening, must rise up: Mag. m.[1]
— — — dry cough, wakening and not ceasing until he sits up in bed and passes flatus upward and downward: *Sang.*[7]
— — — dry cough, night, has to sit up and hold chest with both hands: Natr. s.[7]
— — — cough almost only when first lying down day or evening; was obliged to sit up and cough it out, when had rest: CON.[1]
— — — must sit up in bed and hold head with both hands, on account of violent cough: Nicc.[14]
— — — must sit up with cough and sweat: Eupion.[1]
— — — cough at night, must sit up to raise sputa: Ferr.[7]
— — — night-cough; must sit up as soon as cough commences: *Ars.*[7]
— — — oppression of chest during cough, must sit up in bed: *Aralia*, Coc. c.—10.

